# Supplementary material for: Spatiotemporal integration of contextual and sensory information within the cortical hierarchy in human pain experience
Source: PLoS Biol. 2024 Nov 13;22(11):e3002910. doi: 10.1371/journal.pbio.3002910 (PMC11602096; doi:10.1371/journal.pbio.3002910)
Supplement: S1 Fig — (A) The pain calibration task was conducted to determine individualized stimulus intensity levels to match the levels of subjective pain experience across individuals. Each dot represents the temperature calibrated for an individual, corresponding to each stimulus intensity level. The mean temperatures (standard deviation) for each stimulus level are as follows: 44.49°C (1.34) for LV1, 45.25°C (1.30) for LV2, 46.02°C (1.27) for LV3, 46.78°C (1.29) for LV4, and 47.47°C (1.26) for LV5. One-way analysis of variance results revealed a significant main effect of the fitted stimulus intensity, F (4, 290) = 49.02, p = 1.786e-31. (B) The distribution of the R2 coefficients of the final linear regression models from the pain calibration task. (C) Each line represents the range of calibrated stimulus intensities for each participant, and dots at the end of lines indicate LV1 and LV5 stimulus intensity. The order of participants is sorted by the temperature of LV1. Data from 59 participants who passed the pain calibration task were displayed in this figure. The underlying data for S1 Fig can be found in S1 Data. (DOCX) [file pbio.3002910.s002.docx]

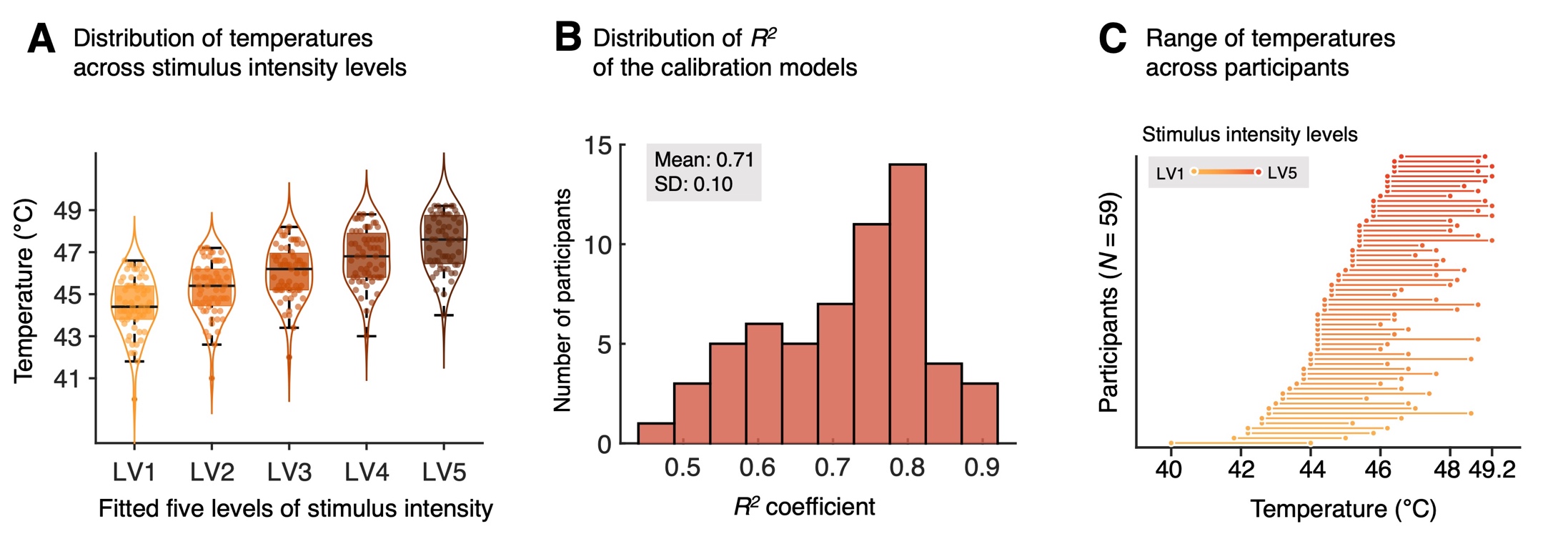


**S1 Fig. Results of pain calibration task. (A)** The pain calibration task was conducted to determine individualized stimulus intensity levels to match the levels of subjective pain experience across individuals. Each dot represents the temperature calibrated for an individual, corresponding to each stimulus intensity level. The mean temperatures (standard deviation) for each stimulus level are as follows: 44.49°C (1.34) for LV1, 45.25°C (1.30) for LV2, 46.02°C (1.27) for LV3, 46.78°C (1.29) for LV4, and 47.47 °C (1.26) for LV5. One-way analysis of variance results revealed a significant main effect of the fitted stimulus intensity, *F* (4, 290) = 49.02, *p* = 1.786e-31. **(B)** The distribution of the *R*^2^ coefficients of the final linear regression models from the pain calibration task. **(C)** Each line represents the range of calibrated stimulus intensities for each participant, and dots at the end of lines indicate LV1 and LV5 stimulus intensity. The order of participants is sorted by the temperature of LV1. Data from 59 participants who passed the pain calibration task were displayed in this figure. The underlying data for S1 Fig can be found in S1 Data.
