# Supplementary material for: Spatiotemporal integration of contextual and sensory information within the cortical hierarchy in human pain experience
Source: PLoS Biol. 2024 Nov 13;22(11):e3002910. doi: 10.1371/journal.pbio.3002910 (PMC11602096; doi:10.1371/journal.pbio.3002910)
Supplement: S2 Fig — Thick and solid lines represent the group average of rating trajectory, while dotted lines represent the within-subject standard error of the mean (SEM). Thin lines illustrate individual averages of rating trajectories. Different colors correspond to experimental conditions. (DOCX) [file pbio.3002910.s003.docx]

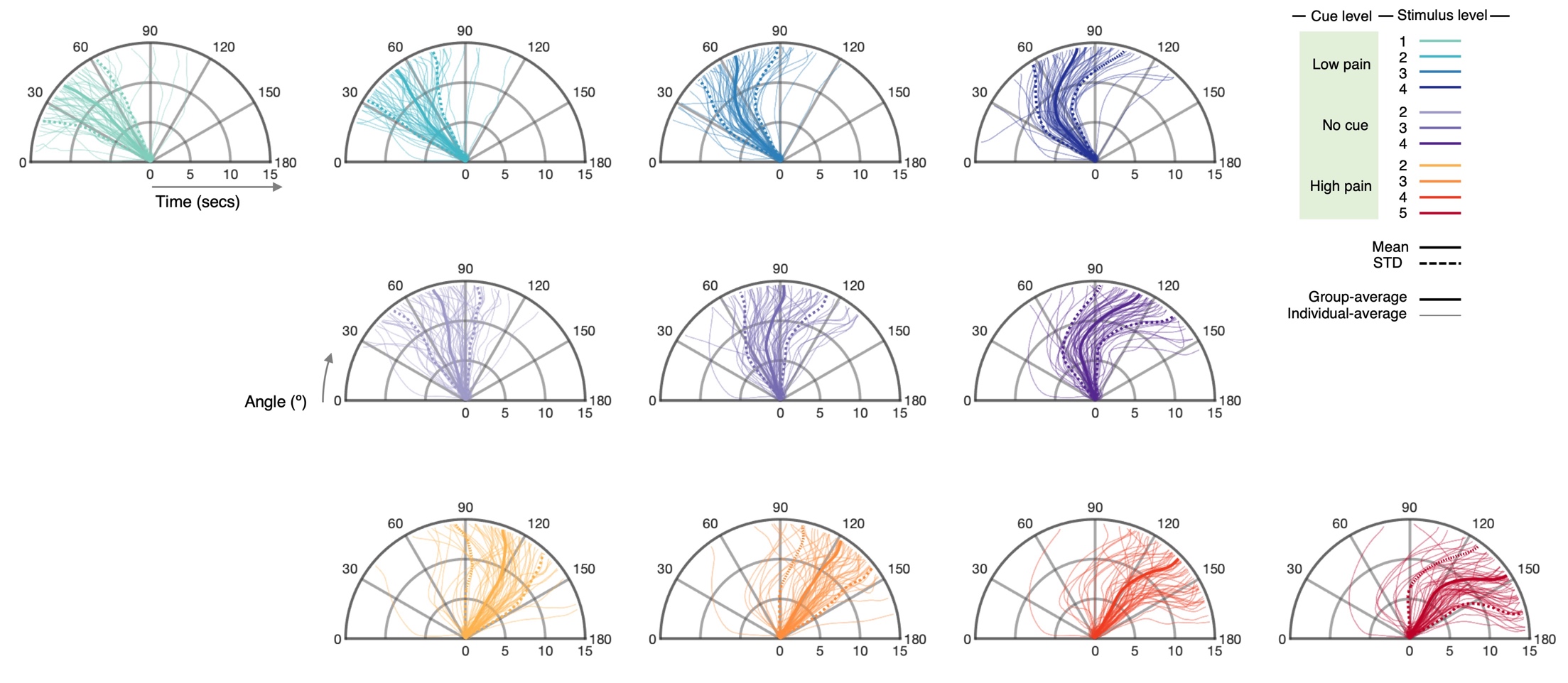


**S2 Fig. Average trajectory of continuous pain prediction ratings for all experimental conditions.** Thick and solid lines represent the group average of rating trajectory, while dotted lines represent the within-subject standard error of the mean (s.e.m.). Thin lines illustrate individual averages of rating trajectories. Different colors correspond to experimental conditions.
