# Supplementary material for: Spatiotemporal integration of contextual and sensory information within the cortical hierarchy in human pain experience
Source: PLoS Biol. 2024 Nov 13;22(11):e3002910. doi: 10.1371/journal.pbio.3002910 (PMC11602096; doi:10.1371/journal.pbio.3002910)
Supplement: S3 Fig — The conjunction map shows the spatial overlap and unique areas involved in the cue and stimulus mediation. Regions colored in cyan and pink denote brain areas associated with the mediation of cue and stimulus effects, respectively. These are thresholded at voxel-wise FDR q < 0.05 with cluster extent k > 5. The areas where these 2 mediation maps overlap are colored in purple. (DOCX) [file pbio.3002910.s004.docx]

**
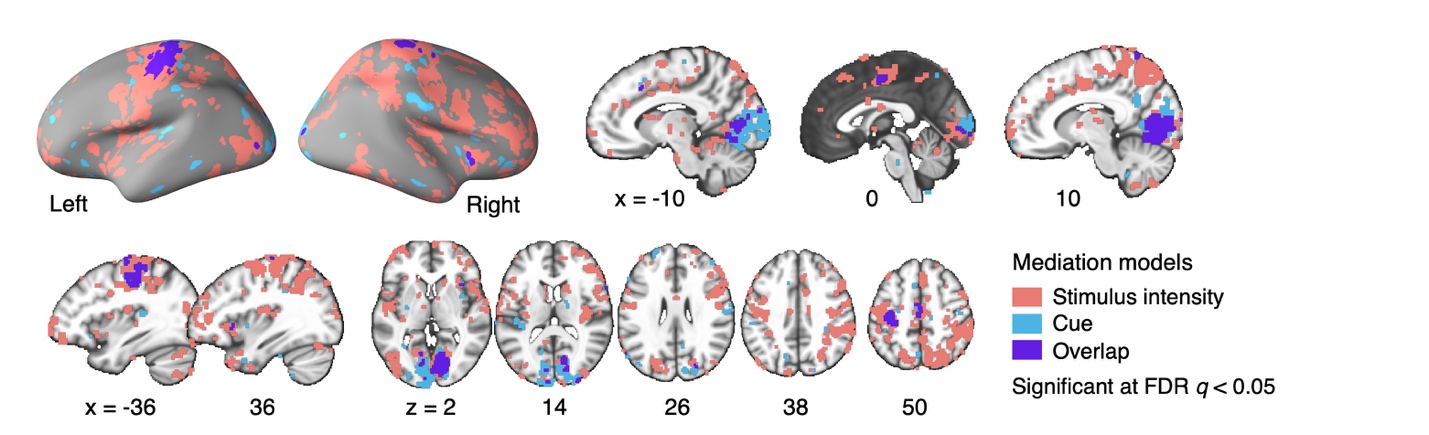
**

**S3 Fig. Conjunction of the cue and the stimulus mediation maps.** The conjunction map shows the spatial overlap and unique areas involved in the cue and stimulus mediation. Regions colored in cyan and pink denote brain areas associated with the mediation of cue and stimulus effects, respectively. These are thresholded at voxel-wise FDR *q* < 0.05 with cluster extent *k* > 5. The areas where these two mediation maps overlap are colored in purple.
