# Supplementary material for: Spatiotemporal integration of contextual and sensory information within the cortical hierarchy in human pain experience
Source: PLoS Biol. 2024 Nov 13;22(11):e3002910. doi: 10.1371/journal.pbio.3002910 (PMC11602096; doi:10.1371/journal.pbio.3002910)
Supplement: S4 Fig — (A) A schematic diagram that illustrates the step-by-step procedure to identify the temporal domains and brain mediators through independent component analysis (ICA). The temporal mediation result matrices (p-values for Path a×b) served as the input for the ICA. We eliminated uninterpretable time domains from analyses, including the mediation effect of later brain activity on earlier ratings or the influence of stimulus intensity on ratings preceding thermal stimulation. These excluded time domains appear as blank in the result matrices. To make the input matrix, we first vectorized the mediation result matrices (i.e., p-values for Path a×b) for each voxel and converted them into -log10(p). Next, we concatenated these vectors across all voxels, thereby aggregating the data from the whole brain. This resulted in matrices with dimensions of 880 or 602 by 24,860. We then obtained temporal and spatial component weights using the GIFToolbox with the fastICA algorithm (# of components = 5). The analysis yielded 2 sets of 5 components, one for spatial weights (24,860 [# of voxels] × 5 [# of components]) and the other for temporal weights (880 or 602 [# of temporal mapping] × 5 [# of components]). To identify the brain mediators and relevant temporal domains for each component, we first thresholded the temporal weights with the top 2.5 percentile. The resulting 5 thresholded temporal weights could be described using a river plot connecting the brain activity and pain ratings. We then identified brain mediators related to these temporal domains based on the following 3 criteria: (1) voxels that survived the FDR correction for multiple comparisons at q < 0.05; (2) regions with at least 5 contiguous voxels; and (3) the survived voxels should cover at least 5% of the defined temporal domain. (B, C) The matrices and brain maps represent the spatial weights and temporal components from the ICA analysis. Warm vs. cool colors represent high vs. low component weights. The areas encl [file pbio.3002910.s005.docx]

**
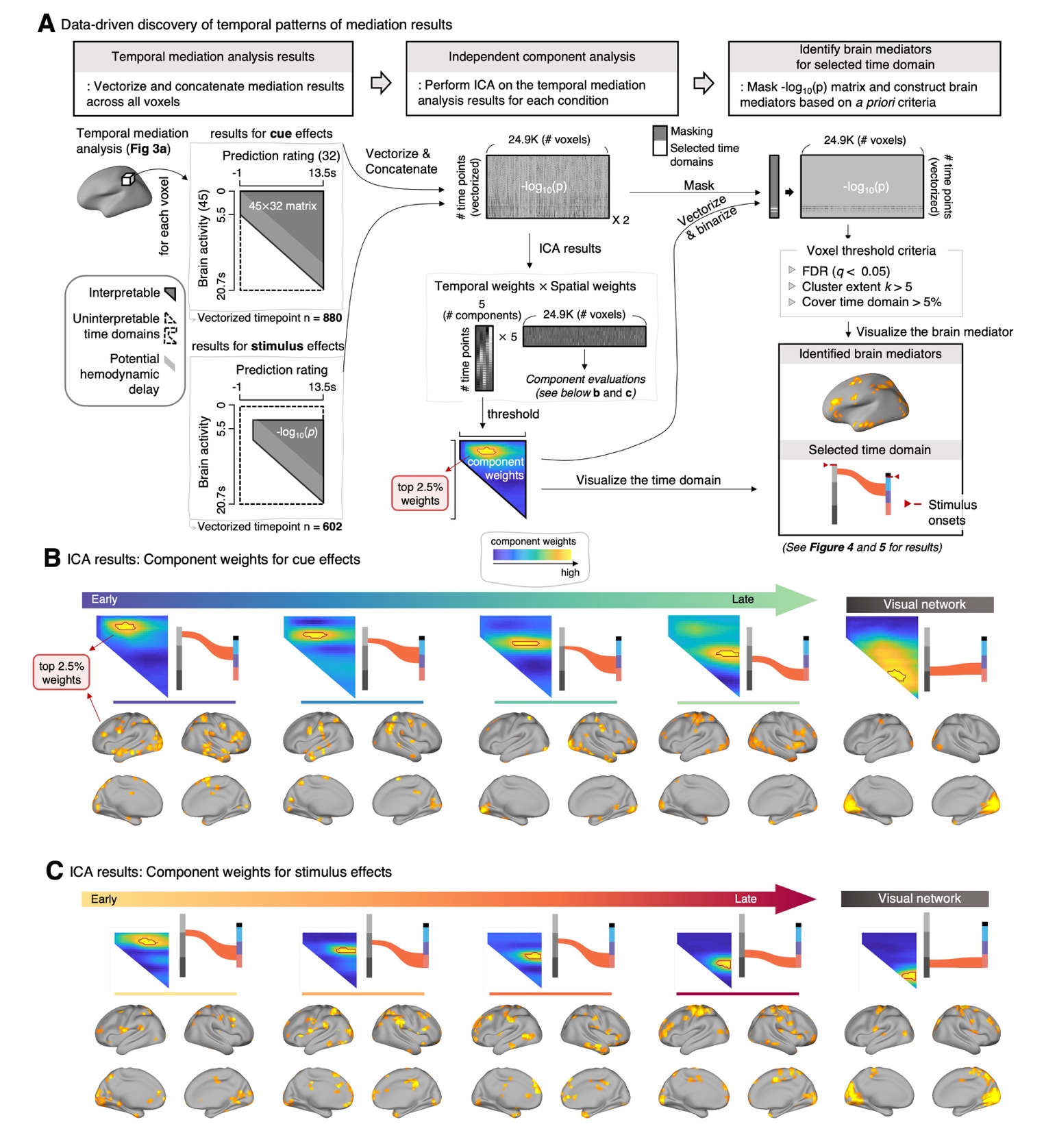

S4 Fig. Independent Component Analysis scheme for the temporal mediation analysis results.**

**(A)** A schematic diagram that illustrates the step-by-step procedure to identify the temporal domains and brain mediators through independent component analysis (ICA). The temporal mediation result matrices (*p*-values for Path *a*×*b*) served as the input for the ICA. We eliminated uninterpretable time domains from analyses, including the mediation effect of later brain activity on earlier ratings or the influence of stimulus intensity on ratings preceding thermal stimulation. These excluded time domains appear as blank in the result matrices. To make the input matrix, we first vectorized the mediation result matrices (i.e., *p*-values for Path *a*×*b*) for each voxel and converted them into -log10(*p*). Next, we concatenated these vectors across all voxels, thereby aggregating the data from the whole brain. This resulted in matrices with dimensions of 880 or 602 by 24,860. We then obtained temporal and spatial component weights using the GIFToolbox with the fastICA algorithm (# of components = 5).

The analysis yielded two sets of five components, one for spatial weights (24,860 [# of voxels]× 5 [# of components]) and the other for temporal weights (880 or 602 [# of temporal mapping] × 5 [# of components]). To identify the brain mediators and relevant temporal domains for each component, we first thresholded the temporal weights with the top 2.5 percentile. The resulting five thresholded temporal weights could be described using a river plot connecting the brain activity and pain ratings. We then identified brain mediators related to these temporal domains based on the following three criteria: 1) voxels that survived the FDR correction for multiple comparisons at *q* < 0.05, 2) regions with at least five contiguous voxels, and 3) the survived voxels should cover at least 5% of the defined temporal domain.

**(B-C)** The matrices and brain maps represent the spatial weights and temporal components from the ICA analysis. Warm vs. cool colors represent high vs. low component weights. The areas enclosed by the red outlines represent the top 2.5% of the weights. One of the identified components was excluded from further analysis due to its strong association with the visual network (right panel), potentially reflecting task-related processes
