# Supplementary material for: Spatiotemporal integration of contextual and sensory information within the cortical hierarchy in human pain experience
Source: PLoS Biol. 2024 Nov 13;22(11):e3002910. doi: 10.1371/journal.pbio.3002910 (PMC11602096; doi:10.1371/journal.pbio.3002910)
Supplement: S5 Fig — (A) Participants completed a simple motor task over 2 runs. They were instructed to move an orange dot to specified target locations on a semicircular rating scale using an MR-compatible joystick. Each run consisted of 21 trials, with each trial including an inter-trial interval of 3–7 s and 6 s for moving the dot. (B) Correlations between the target and actual final positions of the orange dot. Each regression line represents the relationship between the target and actual final positions for individual participants. The angle of 0 to 180° was normalized to a 0 to 1 scale. (C) (Left) Brain maps displaying activation patterns during the simple motor task (FDR q < 0.0001 and a cluster size k < 2 voxels). (Right) Common brain mediators for both cue and stimulus intensity from Fig 6B. Red dashed circles highlight the somatomotor and visual cortex regions that served as common brain mediators for both cue and stimulus effects. The underlying data for S5B Fig can be found in S1 Data. (DOCX) [file pbio.3002910.s006.docx]

**
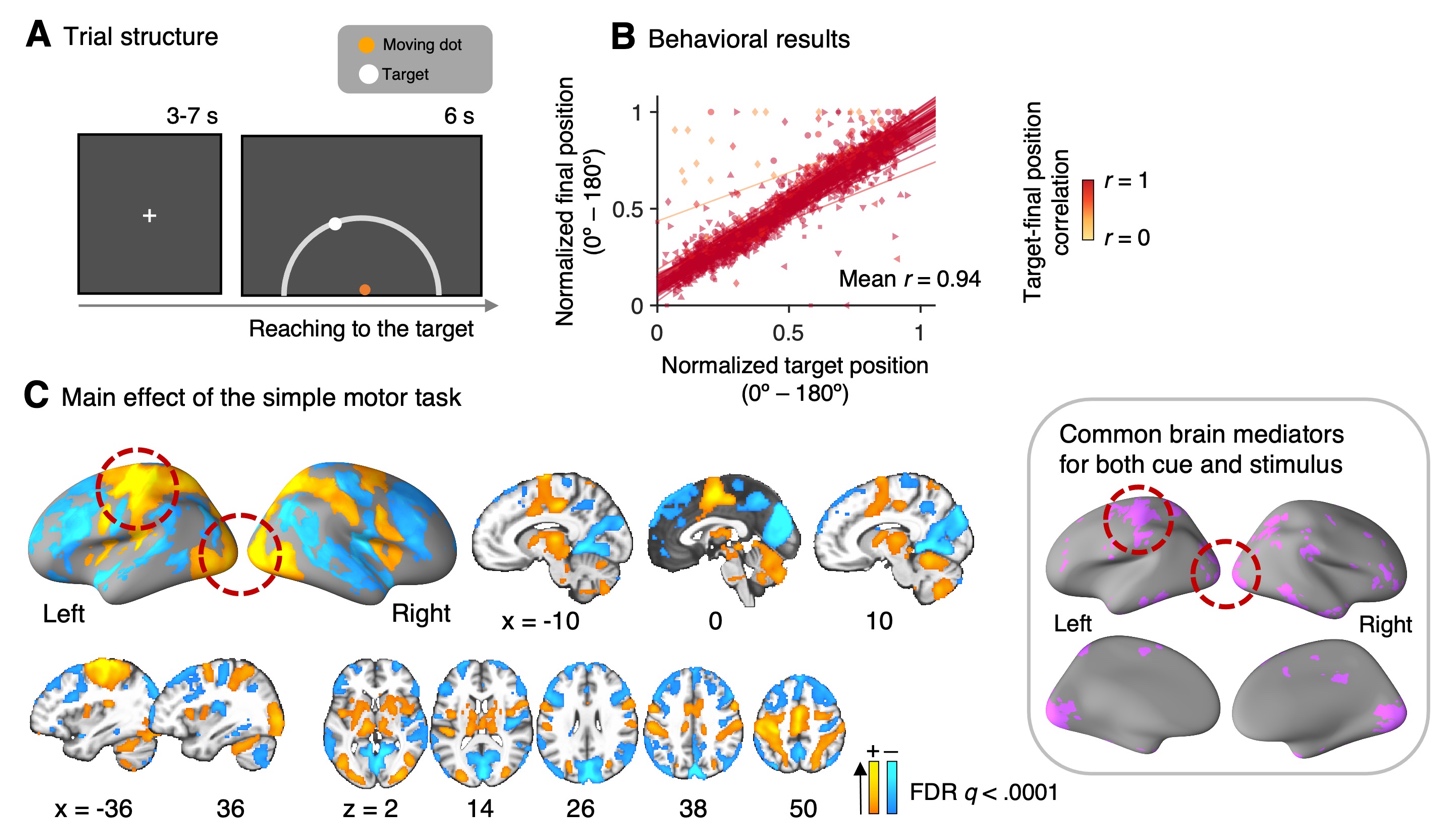
**

**S5 Fig. Simple motor task. (A)** Participants completed a simple motor task over two runs. They were instructed to move an orange dot to specified target locations on a semi-circular rating scale using an MR-compatible joystick. Each run consisted of 21 trials, with each trial including an inter-trial interval of 3-7 seconds and 6 seconds for moving the dot. **(B)** Correlations between the target and actual final positions of the orange dot. Each regression line represents the relationship between the target and actual final positions for individual participants. The angle of 0 to 180 º was normalized to a 0 to 1 scale. **(C)** (Left) Brain maps displaying activation patterns during the simple motor task (FDR *q* < 0.0001 and a cluster size *k* < 2 voxels). (Right) Common brain mediators for both cue and stimulus intensity from **Fig 6B**. Red dashed circles highlight the somatomotor and visual cortex regions that served as common brain mediators for both cue and stimulus effects. The underlying data for S5B Fig can be found in S1 Data.
