# Supplementary material for: Spatiotemporal integration of contextual and sensory information within the cortical hierarchy in human pain experience
Source: PLoS Biol. 2024 Nov 13;22(11):e3002910. doi: 10.1371/journal.pbio.3002910 (PMC11602096; doi:10.1371/journal.pbio.3002910)
Supplement: S6 Fig — To investigate the relationship between continuous pain prediction ratings at each time window (comprising 32 TR-level bins) and overall pain ratings, we averaged the continuous pain ratings within each TR window. We then calculated Pearson’s correlation between these time-averaged continuous ratings and the overall pain ratings. The resulting plot demonstrates an overall increase in correlations throughout the trial. Analysis of variance (ANOVA) results revealed a significant time effect, F(29, 1,740) = 62.35, p = 1.60276e-244. This finding implies that the later stages of the continuous pain prediction ratings provide more information into the overall pain ratings compared to the earlier and middle stages. The underlying data for S6 Fig can be found in S1 Data. (DOCX) [file pbio.3002910.s007.docx]

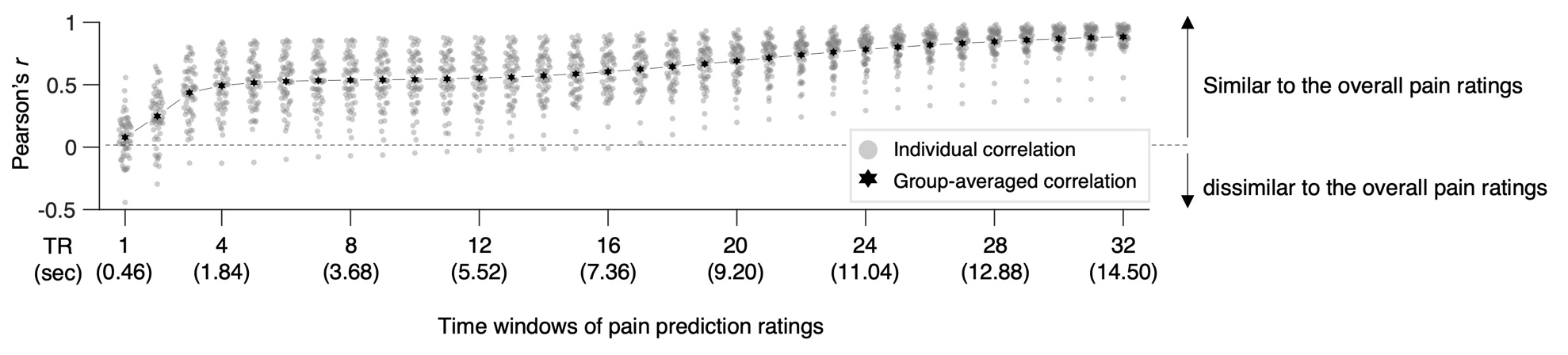


**S6 Fig. Correlations between the continuous pain prediction ratings and the overall pain ratings.** To investigate the relationship between continuous pain prediction ratings at each time window (comprising 32 TR-level bins) and overall pain ratings, we averaged the continuous pain ratings within each TR window. We then calculated Pearson's correlation between these time-averaged continuous ratings and the overall pain ratings. The resulting plot demonstrates an overall increase in correlations throughout the trial. Analysis of variance (ANOVA) results revealed a significant time effect, *F*(29,1740) = 62.35, *p* = 1.60276e-244. This finding implies that the later stages of the continuous pain prediction ratings provide more information into the overall pain ratings compared to the earlier and middle stages. The underlying data for S6 Fig can be found in S1 Data.
