# Supplementary material for: Spatiotemporal integration of contextual and sensory information within the cortical hierarchy in human pain experience
Source: PLoS Biol. 2024 Nov 13;22(11):e3002910. doi: 10.1371/journal.pbio.3002910 (PMC11602096; doi:10.1371/journal.pbio.3002910)
Supplement: S7 Fig — Each row of brain maps corresponds to temporal domains of the (A) cue and (B) stimulus mediation. Brain regions where Paths a and b are both positive are depicted in red, and regions where both Paths a and b are negative are depicted in blue. Radial plots show the relative proportions of the number of overlapping voxels between the thresholded mediation maps with positive (red) and negative (blue) Paths a and b and each of the large-scale networks (or regions) given the total number of voxels within each network (or region). VA, ventral attention network; DA, dorsal attention network; SM, somatomotor network; BS, brainstem; HC/Amy, hippocampus and amygdala; TH, Thalamus; DM, default mode network; FP, frontoparietal network; LIM, limbic network. (DOCX) [file pbio.3002910.s008.docx]

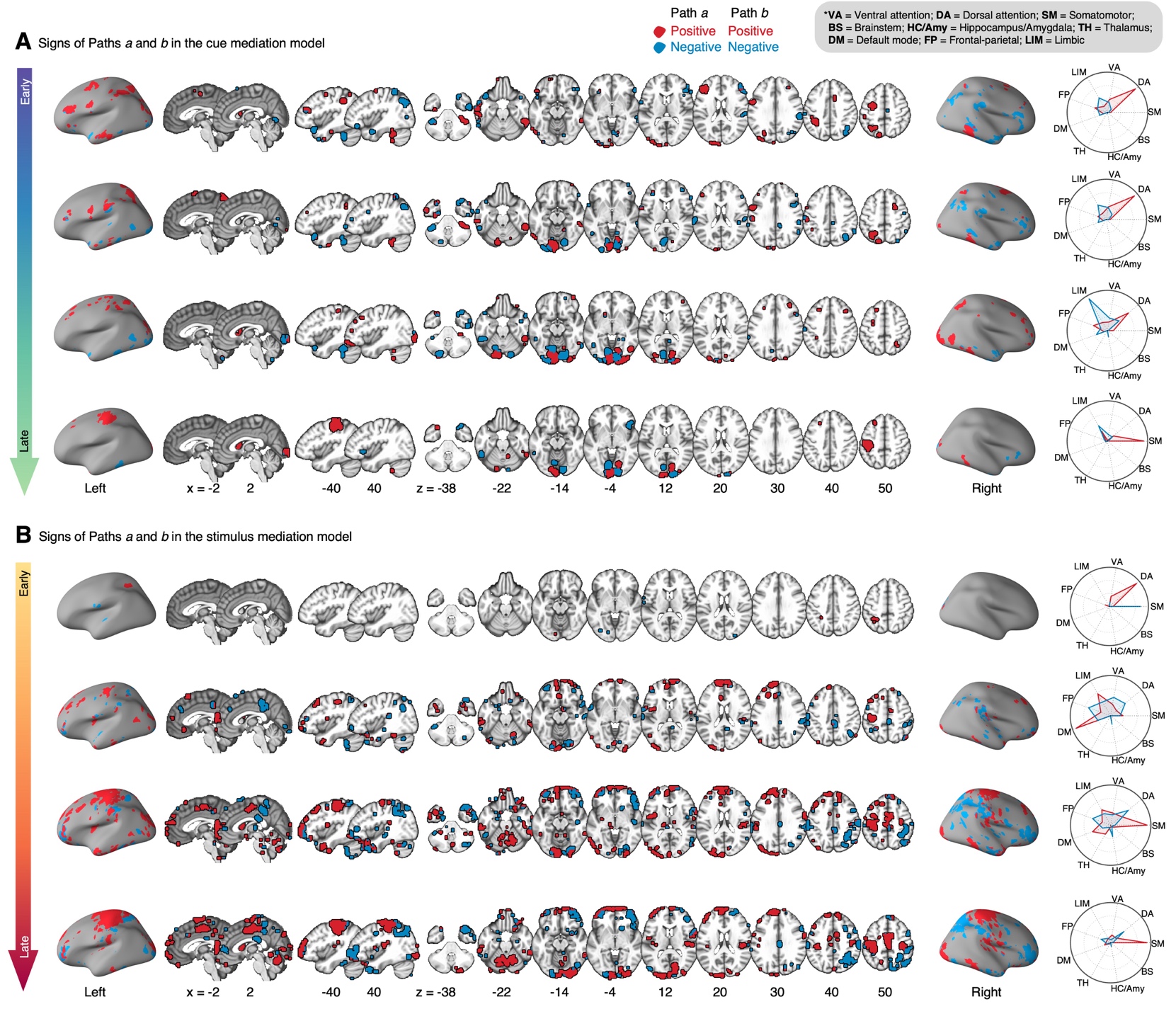


**S7 Fig. Signs of Paths *a* and *b* of brain mediators for cue and stimulus intensity effects.** Each row of brain maps corresponds to temporal domains of the **(A)** cue and **(B)** stimulus mediation. Brain regions where Paths *a* and *b* are both positive are depicted in red, and regions where both Paths *a* and *b* are negative are depicted in blue. Radial plots show the relative proportions of the number of overlapping voxels between the thresholded mediation maps with positive (red) and negative (blue) Paths *a* and *b* and each of the large-scale networks (or regions) given the total number of voxels within each network (or region). VA, ventral attention network; DA, dorsal attention network; SM, somatomotor network; BS, brainstem; HC/Amy, hippocampus and amygdala; TH, Thalamus; DM, default mode network; FP, frontoparietal network; LIM, limbic network.
