# Supplementary material for: Spatiotemporal integration of contextual and sensory information within the cortical hierarchy in human pain experience
Source: PLoS Biol. 2024 Nov 13;22(11):e3002910. doi: 10.1371/journal.pbio.3002910 (PMC11602096; doi:10.1371/journal.pbio.3002910)
Supplement: S8 Fig — To assess the robustness of the temporal mediation analysis results presented in Figs 4 and 5, we performed the same analysis with different hyperparameters and evaluated the spatial similarity with the original mediation maps. (A) Analysis Pipeline (left): We obtained brain mediation maps using the same analysis pipeline from the original results, modifying only 1 hyperparameter. We then calculated the spatial similarity between thresholded maps using Dice coefficients. The spatial similarity was visualized via multidimensional scaling (MDS) in 2 dimensions. In the resulting MDS plots, the distance between brain mediators represents their similarity, with greater similarity indicated by closer proximity. Example results (middle): For instance, testing a new threshold for temporal weights yields 5 new mediation maps, one for each component. We can compute the spatial similarity among 10 mediation maps—5 from the original results and 5 from the new analysis results. Focusing on the orange box cases, we examine the relationship between the first mediation map from the original results and the first and fifth mediation maps from the new analysis results. A shorter distance in the MDS plot reflects greater similarity between the original and first new mediation maps, while a larger distance indicates lower similarity between the original and fifth new mediation maps. Figure legend for (C) (right): The color scheme used in Figs 4 and 5 was applied to represent mediation maps for cue and stimulus intensity. The dark gray dot signifies a mediation map from the visual cortex-dominant component. The numbers in the MDS plots denote new mediation maps derived from analyses with varying hyperparameters. (B) We varied the number of ICA components, the percentile of temporal component weights, and the coverage of the defined temporal domain for voxel thresholding one at a time, while keeping all other analysis parameters consistent with Figs 4 and 5. (C) Spatial similarity result [file pbio.3002910.s009.docx]

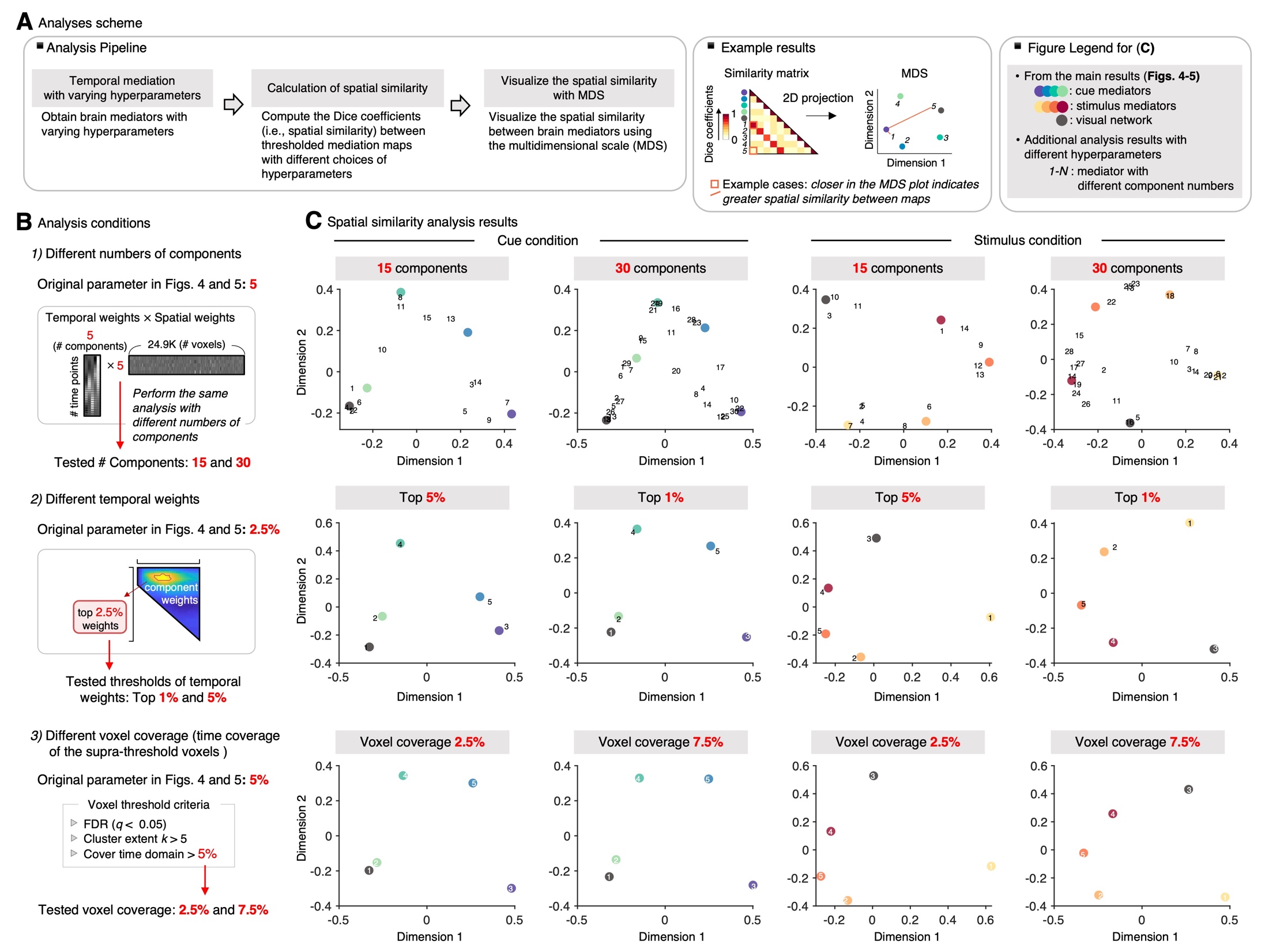


**S8 Fig. Temporal mediation analysis with different hyperparameters.** To assess the robustness of the temporal mediation analysis results presented in **Figs 4** and **5**, we performed the same analysis with different hyperparameters and evaluated the spatial similarity with the original mediation maps.

**(A)** Analysis Pipeline (left): We obtained brain mediation maps using the same analysis pipeline from the original results, modifying only one hyperparameter. We then calculated the spatial similarity between thresholded maps using Dice coefficients. The spatial similarity was visualized via multidimensional scaling (MDS) in two dimensions. In the resulting MDS plots, the distance between brain mediators represents their similarity, with greater similarity indicated by closer proximity. Example results (middle): For instance, testing a new threshold for temporal weights yields five new mediation maps, one for each component. We can compute the spatial similarity among 10 mediation maps—five from the original results and five from the new analysis results. Focusing on the orange box cases, we examine the relationship between the first mediation map from the original results and the first and fifth mediation maps from the new analysis results. A shorter distance in the MDS plot reflects greater similarity between the original and first new mediation maps, while a larger distance indicates lower similarity between the original and fifth new mediation maps. Figure legend for C (right): The color scheme used in **Figs 4** and **5** was applied to represent mediation maps for cue and stimulus intensity. The dark gray dot signifies a mediation map from the visual cortex-dominant component. The numbers in the MDS plots denote new mediation maps derived from analyses with varying hyperparameters.

**(B)** We varied the number of ICA components, the percentile of temporal component weights, and the coverage of the defined temporal domain for voxel thresholding one at a time, while keeping all other analysis parameters consistent with **Figs 4** and **5**.

**(C)** Spatial similarity results: MDS was used to project the spatial similarities between brain mediators from the main and additional analyses into a 2-dimensional space. Colored circles represent the mediators from the original results in **Figs 4** and **5**, while number markers correspond to the results from new analyses with different hyperparameters. Mediation maps with altered temporal weights and voxel coverages generally aligned with the original results. Even when more components were tested, many new brain mediation maps exhibited high similarity to the original maps. Overall, the additional analyses with varying hyperparameters produced brain mediator patterns largely consistent with the original findings.
