# Supplementary material for: Spatiotemporal integration of contextual and sensory information within the cortical hierarchy in human pain experience
Source: PLoS Biol. 2024 Nov 13;22(11):e3002910. doi: 10.1371/journal.pbio.3002910 (PMC11602096; doi:10.1371/journal.pbio.3002910)
Supplement: S9 Fig — We conducted a region-of-interest (ROI)-based approach to complement the results of the whole-brain temporal mediation analysis results. For a detailed description of the ROI-based temporal mediation analysis, please refer to the Materials and methods. (A) Based on prior literature [13,16,66], we selected 17 a priori ROIs known for their roles in pain processing. These include areas involved in nociceptive pain processing [66] (e.g., left and right thalamus, right second somatosensory area, and left and right dorsal-posterior insula cortex), regions associated with self-regulatory strategies in response to pain [16] (e.g., ventromedial prefrontal cortex and nucleus accumbens), and areas linked to social information processing related to pain [13] (e.g., pregenual anterior cingulate cortex, orbitofrontal cortex, left and right ventrolateral prefrontal cortex, left supramarginal gyrus, medial frontal gyrus, intraparietal sulcus, and right dorsolateral prefrontal cortex). (B, C) River plots illustrate the ROI-based temporal mediation analysis results. The results were categorized by brain mediation timing—“early” and “late.” In addition, the regions with significance in more than 2 domains were categorized as “multiple.” The plots show the temporal domains significant at a false discovery rate (FDR) q < 0.05, alongside adjacent time domains pruned using 2 more liberal thresholds (p < 0.005 and p < 0.01) to contextualize the results. FDR, false discovery rate; vmPFC, ventromedial prefrontal cortex; pgACC, pregenual anterior cingulate cortex; NAc, nucleus accumbens; dpINS, dorsal-posterior insular; S2, second somatosensory area; OFC, orbitofrontal cortex; vlPFC, ventrolateral prefrontal cortex; SMG, supramarginal gyrus; MFG, medial frontal gyrus; IPS, intraparietal sulcus; dlPFC, dorsolateral prefrontal cortex. (DOCX) [file pbio.3002910.s010.docx]

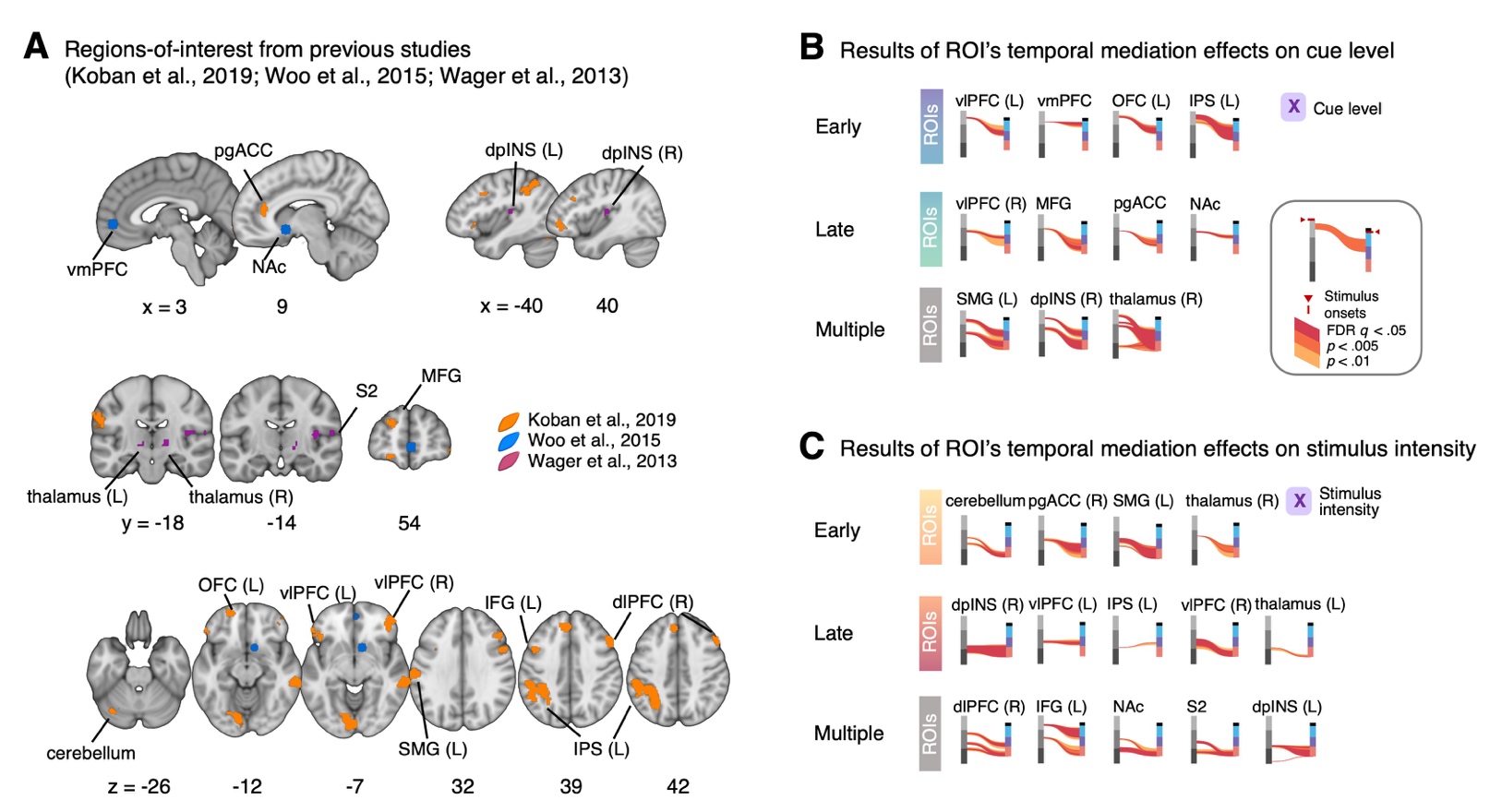


**S9 Fig. Region-of-interest (ROI)-based temporal mediation analysis results.** We conducted a region-of-interest (ROI)-based approach to complement the results of the whole-brain temporal mediation analysis results. For a detailed description of the ROI-based temporal mediation analysis, please refer to the *Materials and Methods*. **(A)** Based on prior literature[13, 16, 66], we selected 17 *a priori* ROIs known for their roles in pain processing. These include areas involved in nociceptive pain processing[66] (e.g., left and right thalamus, right second somatosensory area, and left and right dorsal-posterior insula cortex), regions associated with self-regulatory strategies in response to pain[16] (e.g., ventromedial prefrontal cortex and nucleus accumbens), and areas linked to social information processing related to pain[13] (e.g., pregenual anterior cingulate cortex, orbitofrontal cortex, left and right ventrolateral prefrontal cortex, left supramarginal gyrus, medial frontal gyrus, intraparietal sulcus, and right dorsolateral prefrontal cortex). **(B-C),** River plots illustrate the ROI-based temporal mediation analysis results. The results were categorized by brain mediation timing—‘early’ and ‘late.’ In addition, the regions with significance in more than two domains were categorized as ‘multiple.’ The plots show the temporal domains significant at a false-discovery rate (FDR) *q* < 0.05, alongside adjacent time domains pruned using two more liberal thresholds (*p* < 0.005 and *p* < 0.01) to contextualize the results. Abbreviations: FDR, false-discovery rate; vmPFC, ventromedial prefrontal cortex; pgACC, pregenual anterior cingulate cortex; NAc, nucleus accumbens; dpINS, dorsal-posterior insular; S2, second somatosensory area; OFC, orbitofrontal cortex; vlPFC, ventrolateral prefrontal cortex; SMG, supramarginal gyrus; MFG, medial frontal gyrus; IPS, intraparietal sulcus; dlPFC, dorsolateral prefrontal cortex.
