# Supplementary material for: Spatiotemporal integration of contextual and sensory information within the cortical hierarchy in human pain experience
Source: PLoS Biol. 2024 Nov 13;22(11):e3002910. doi: 10.1371/journal.pbio.3002910 (PMC11602096; doi:10.1371/journal.pbio.3002910)
Supplement: S10 Fig — (A) Participants were thoroughly instructed on how to use the scale before proceeding to the pain calibration task on Day 1. For this, we used a structured instruction script, and the instructions focused primarily on the anchors and corresponding labels of the scale. As noted in the main text, we employed a modified version of the generalized labeled magnitude scale (gLMS [70]) to define the anchors. These anchors included no sensation (0°), weak (18°), moderate (50°), strong (108°), very strong (162°), and strongest imaginable sensation (180°). We emphasized that the label “Weak” marked the point where the sensation began to feel painful. To ensure participants fully understood the instructions, they were asked to explain the scale back to experimenters. These figures show the slide presented for this “explaining-back” procedure. If a participant was unable to explain the scale correctly, we provided the instructions again until they fully grasped the scale. (B) The key aspect of the prediction rating was for participants to continuously report their ongoing estimate of how painful the stimulus would be, indicating their “current” prediction of the pain level. (Top) We showed participants an example of continuous pain prediction ratings to explain the pain prediction rating and how to report it. We instructed them the angle from the start point will be regarded as a current pain prediction. We also instructed that they did not need to click the button to report the prediction continuously, highlighting the difference between the prediction ratings and the intensity ratings. (Bottom) We asked participants to explain what they understood about the pain prediction back to the experimenter. If their understanding was incomplete, the procedure was repeated until they fully grasped it. The English translation of the text was not displayed during the actual experiment. (DOCX) [file pbio.3002910.s011.docx]

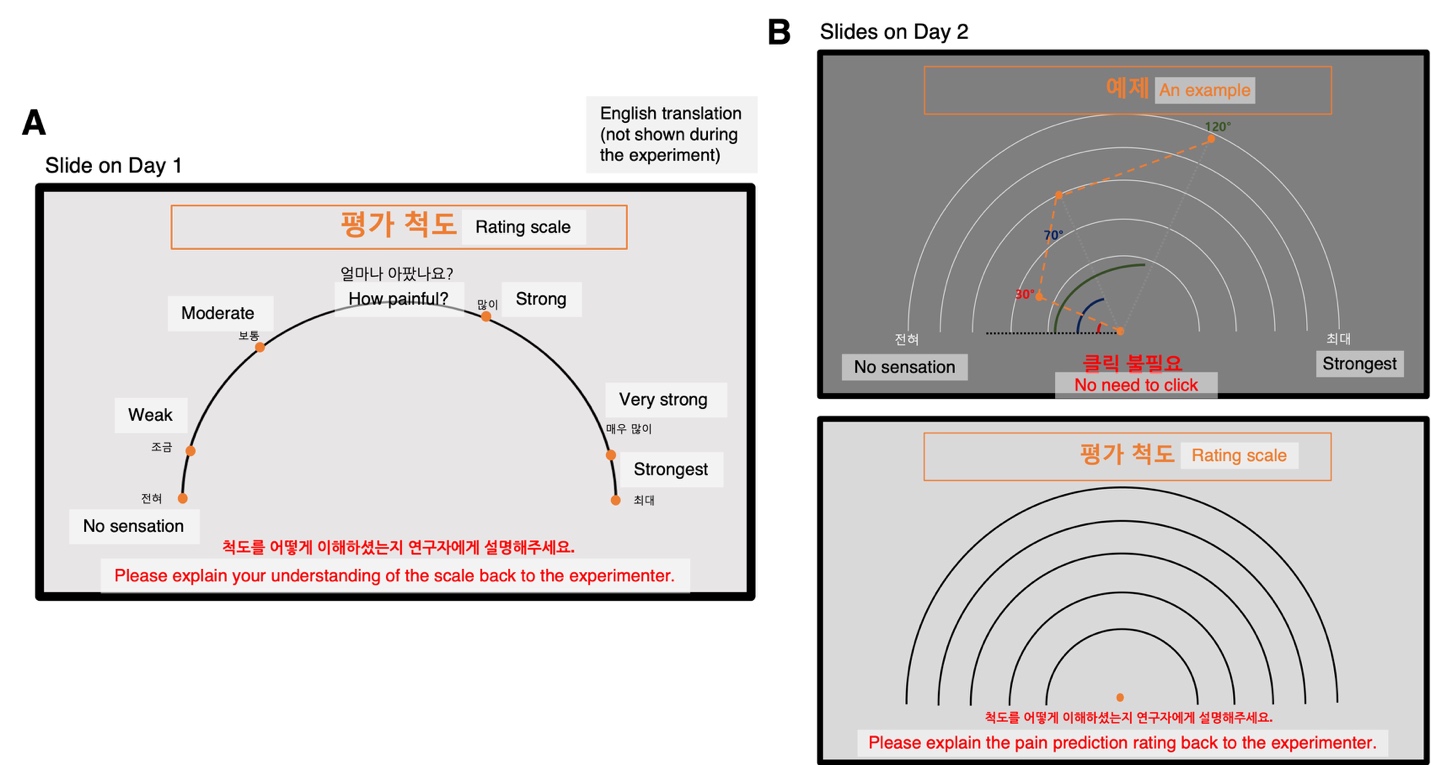


**S10 Fig. The actual slide presented on Day 1 and Day 2 to ensure participants fully understood the rating scale and the pain prediction rating. (A)** Participants were thoroughly instructed on how to use the scale before proceeding to the pain calibration task on Day 1. For this, we used a structured instruction script, and the instructions focused primarily on the anchors and corresponding labels of the scale. As noted in the main text, we employed a modified version of the generalized Labeled Magnitude Scale (gLMS [70]) to define the anchors. These anchors included no sensation (0º), weak (18º), moderate (50º), strong (108º), very strong (162º), and strongest imaginable sensation (180º). We emphasized that the label “Weak” marked the point where the sensation began to feel painful. To ensure participants fully understood the instructions, they were asked to explain the scale back to experimenters. These figures show the slide presented for this “explaining-back” procedure. If a participant was unable to explain the scale correctly, we provided the instructions again until they fully grasped the scale.

**(B)** The key aspect of the prediction rating was for participants to continuously report their ongoing estimate of how painful the stimulus would be, indicating their ‘current’ prediction of the pain level. (top) We showed participants an example of continuous pain prediction ratings to explain the pain prediction rating and how to report it. We instructed them the angle from the start point will be regarded as a current pain prediction. We also instructed that they did not need to click the button to report the prediction continuously, highlighting the difference between the prediction ratings and the intensity ratings. (bottom) We asked participants to explain what they understood about the pain prediction back to the experimenter. If their understanding was incomplete, the procedure was repeated until they fully grasped it. The English translation of the text was not displayed during the actual experiment.
