# Supplementary material for: Spatiotemporal integration of contextual and sensory information within the cortical hierarchy in human pain experience
Source: PLoS Biol. 2024 Nov 13;22(11):e3002910. doi: 10.1371/journal.pbio.3002910 (PMC11602096; doi:10.1371/journal.pbio.3002910)
Supplement: S11 Fig — (A) Schematic overview of trial structure. (B) Example of the continuous pain prediction rating: (Left) The figure shows an example rating trajectory over the 14.5 s during the pain prediction rating period. Starting from the center (i.e., the initial point), participants were asked to move the orange dot anywhere within the screen to report their continuous ratings. We emphasized that it was the angle, not the distance, that reflected participants’ rating, and that they were not required to reach the outer end of the semicircles. The rating trajectory was recorded with the dot’s x and y coordinates, but it was converted to the angle from the left segment of the semicircle base. (Right) The (x, y) coordinates were converted to the angles, which served as online ratings. Only the current dot location represented as the orange dot was visible to participants. (C) Example of the overall pain rating: (Left) The figure shows an example rating trajectory for the overall pain rating. (Right) The dot’s (x, y) coordinates were converted to the angles. Then, we calculated the average of the angles of the last 1 s, which served as the overall pain rating. We made a short video of an example trial (https://youtu.be/tR0LUDaYt38) to illustrate what participants saw in the scanner. (DOCX) [file pbio.3002910.s012.docx]

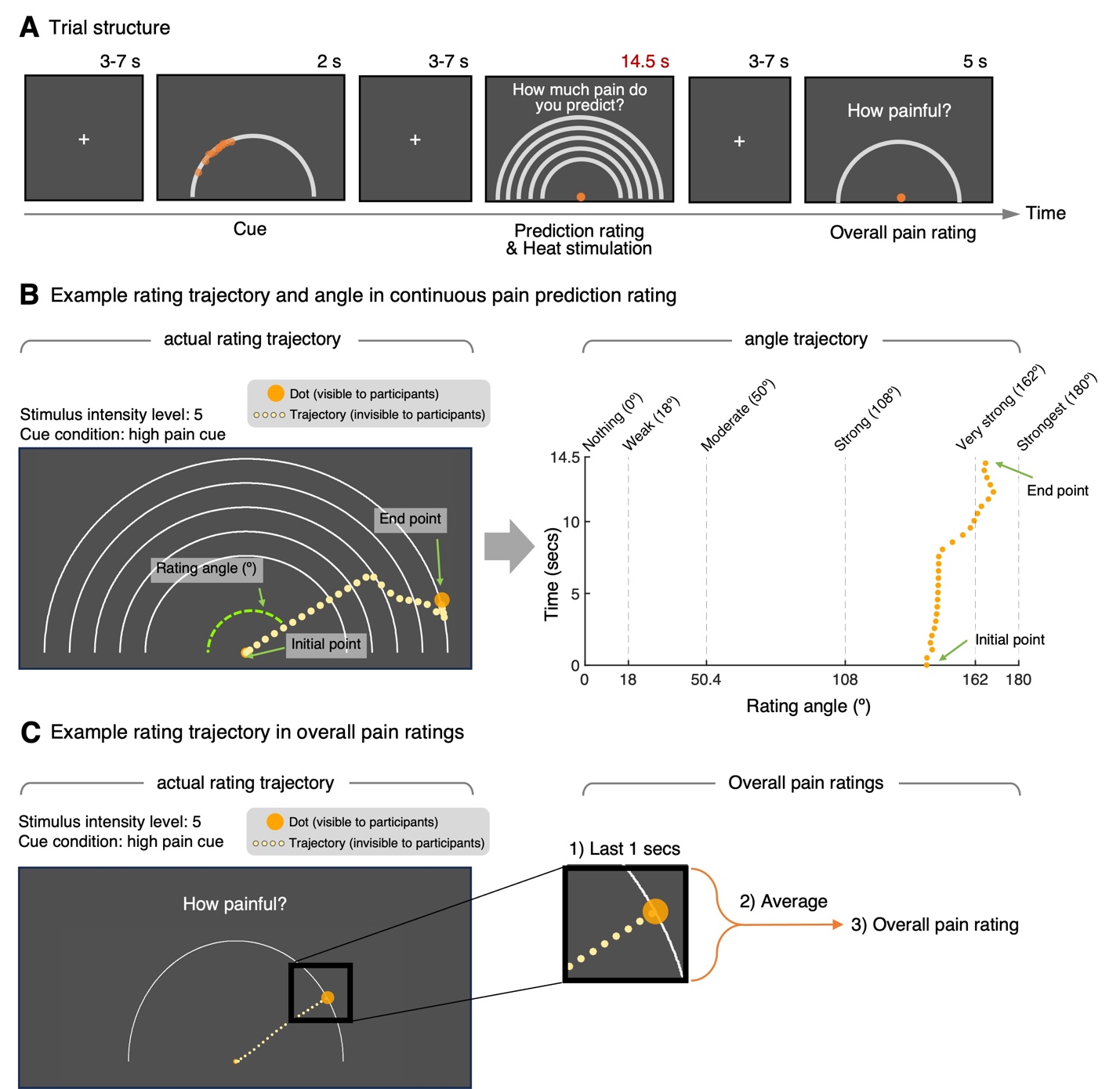


**S11 Fig. Trial structure and rating trajectory examples. (A)** Schematic overview of trial structure. **(B)** Example of the continuous pain prediction rating: (Left) The figure shows an example rating trajectory over the 14.5 seconds during the pain prediction rating period. Starting from the center (i.e., the initial point), participants were asked to move the orange dot anywhere within the screen to report their continuous ratings. We emphasized that it was the angle, not the distance, that reflected participants’ rating, and that they were not required to reach the outer end of the semicircles. The rating trajectory was recorded with the dot’s *x* and *y* coordinates, but it was converted to the angle from the left segment of the semicircle base. (Right) The (*x*, *y*) coordinates were converted to the angles, which served as online ratings. Only the current dot location represented as the orange dot was visible to participants. **(C)** Example of the overall pain rating: (Left) The figure shows an example rating trajectory for the overall pain rating. (Right) The dot’s (*x*, *y*) coordinates were converted to the angles. Then, we calculated the average of the angles of the last 1 second, which served as the overall pain rating. We made a short video of an example trial (<https://youtu.be/tR0LUDaYt38>) to illustrate what participants saw in the scanner.
