# Supplementary material for: Spatiotemporal integration of contextual and sensory information within the cortical hierarchy in human pain experience
Source: PLoS Biol. 2024 Nov 13;22(11):e3002910. doi: 10.1371/journal.pbio.3002910 (PMC11602096; doi:10.1371/journal.pbio.3002910)
Supplement: S12 Fig — We calculated the temporal signal-to-noise ratio (tSNR) using the TR-level data of the prediction task runs. We then averaged the tSNR values across runs and participants. The map shows the group average of the tSNR values. (DOCX) [file pbio.3002910.s013.docx]

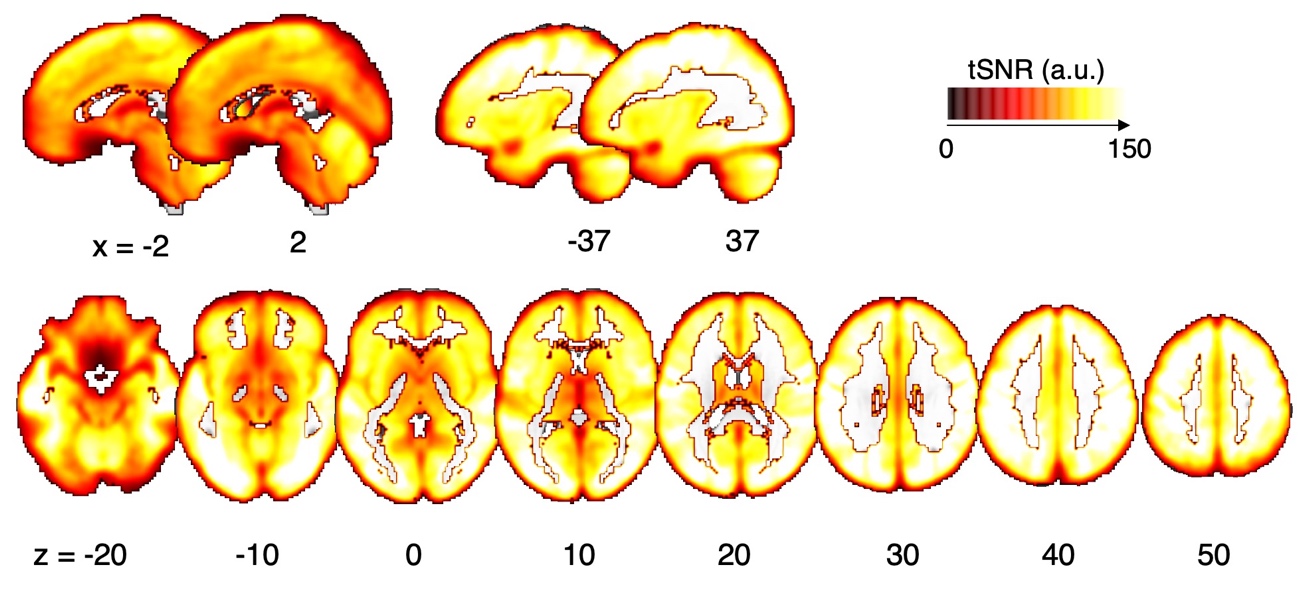


**S12 Fig. Temporal Signal-to-Noise Ratio (tSNR) map.** We calculated the temporal signal-to-noise ratio (tSNR) using the TR-level data of the prediction task runs. We then averaged the tSNR values across runs and participants. The map shows the group average of the tSNR values.
