# Supplementary material for: Spatiotemporal integration of contextual and sensory information within the cortical hierarchy in human pain experience
Source: PLoS Biol. 2024 Nov 13;22(11):e3002910. doi: 10.1371/journal.pbio.3002910 (PMC11602096; doi:10.1371/journal.pbio.3002910)
Supplement: S13 Fig — Principal gradient map diagnostics and resulting map. (A) Scaled Eigenvalues across components. (B) A scatter plot of the first and second connectivity-based principal gradients. Each dot in the scatter plot represents connectivity, and different color represents the relationship between the first and second gradients. The gradient pattern was largely consistent with the original study [33]. (C) A brain map representing the first and second gradients using the same color scheme as the scatter plot in B. The underlying data for S13 Fig can be found in S1 Data. (DOCX) [file pbio.3002910.s014.docx]

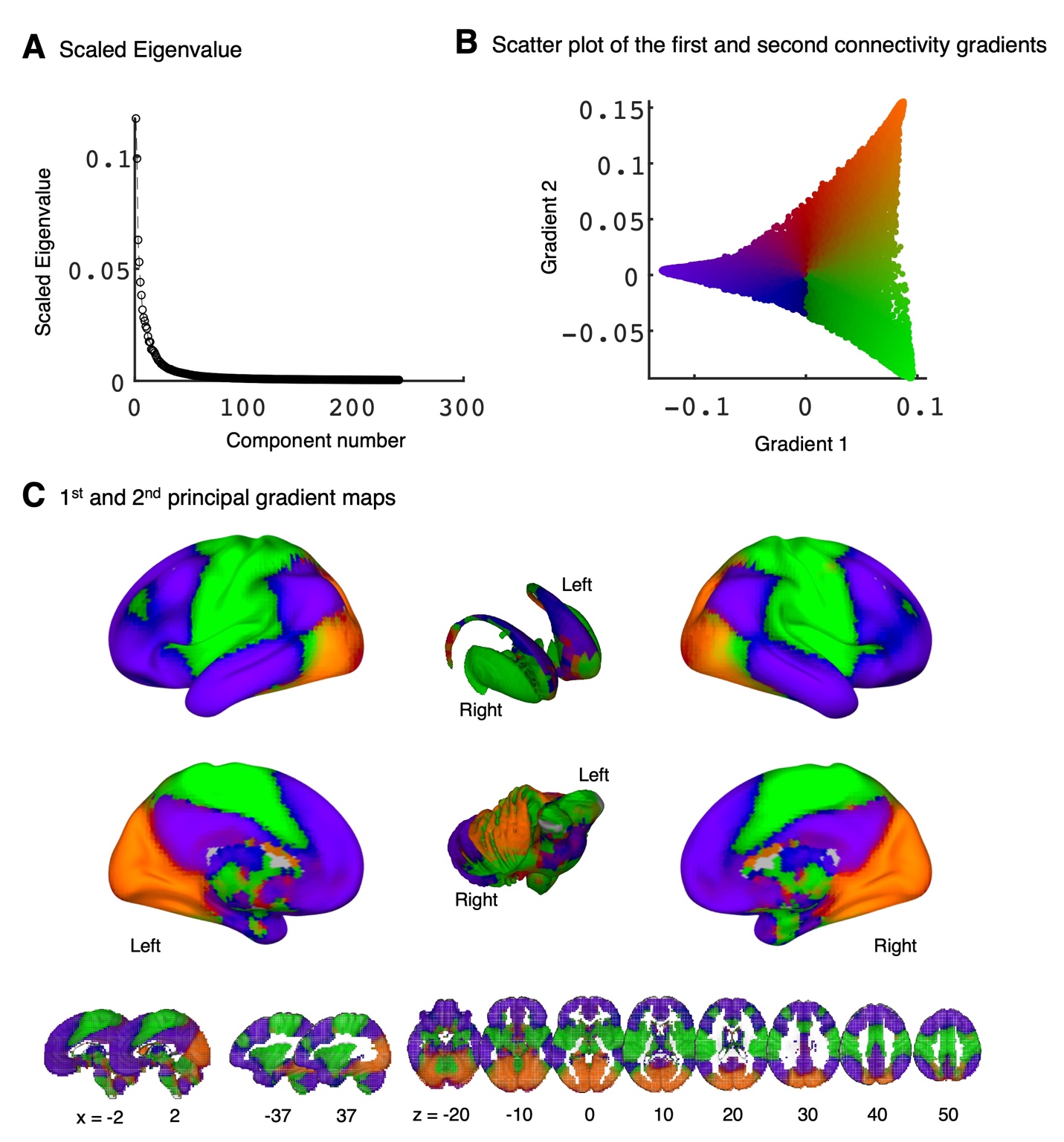


**S13 Fig. Building a volumetric gradient map.** Principal gradient map diagnostics and resulting map. **(A)** Scaled Eigenvalues across components. **(B)** A scatter plot of the 1^st^ and 2^nd^ connectivity-based principal gradients. Each dot in the scatter plot represents connectivity, and different color represents the relationship between the 1^st^ and 2^nd^ gradients. The gradient pattern was largely consistent with the original study[33]. (**C)** A brain map representing the 1^st^ and 2^nd^ gradients using the same color scheme as the scatter plot in **B**. The underlying data for S13 Fig can be found in S1 Data.
