# Supplementary material for: Spatiotemporal integration of contextual and sensory information within the cortical hierarchy in human pain experience
Source: PLoS Biol. 2024 Nov 13;22(11):e3002910. doi: 10.1371/journal.pbio.3002910 (PMC11602096; doi:10.1371/journal.pbio.3002910)
Supplement: S14 Fig — Different colors represent different large-scale networks and subcortical regions. A large-scale functional network includes seven cortical [75], basal ganglia [77], and cerebellum networks [76]. In addition to these large-scale networks, we added thalamus, hippocampus/amygdala, and brainstem [78]. (DOCX) [file pbio.3002910.s015.docx]

**
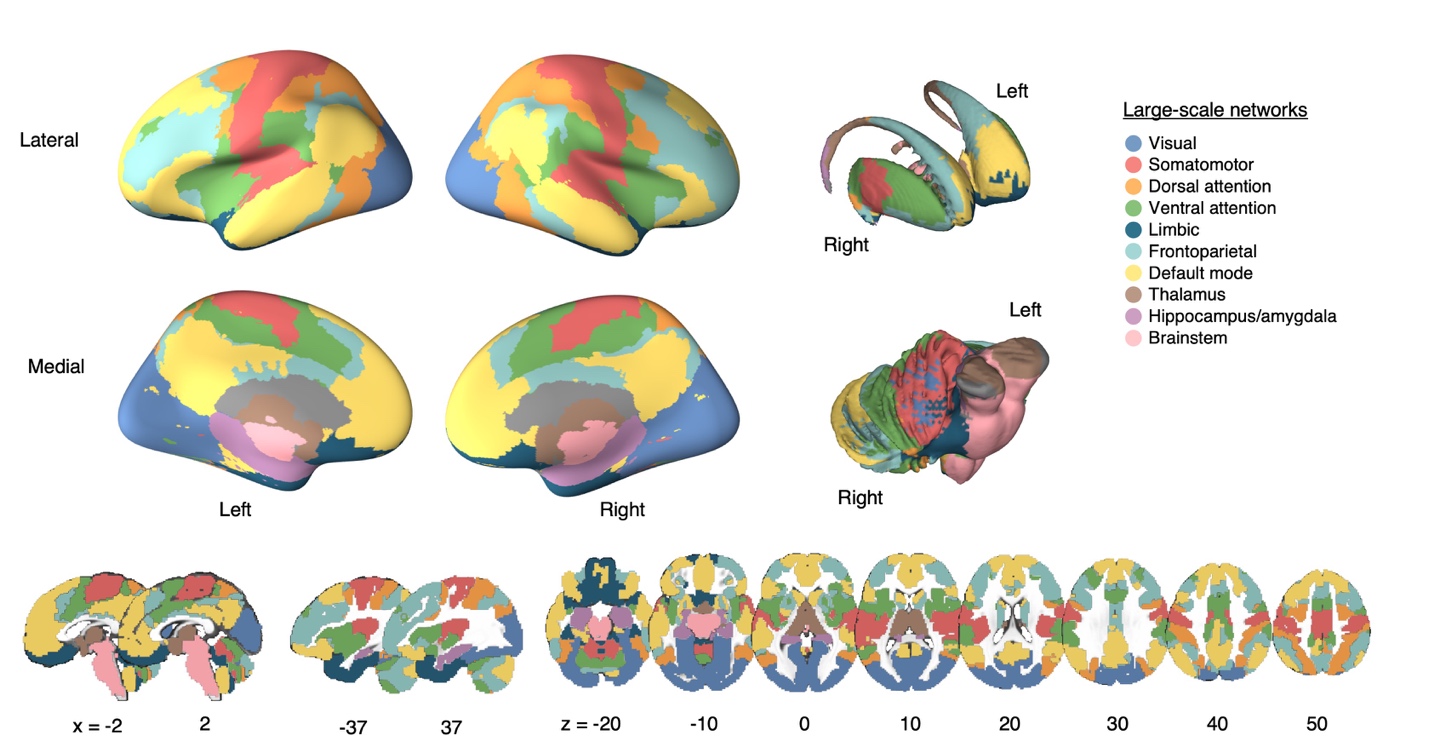
**

**S14 Fig. A large-scale functional brain networks and subcortical regions.** Different colors represent different large-scale networks and subcortical regions. A large-scale functional network includes seven cortical[75], basal ganglia[77], and cerebellum networks[76]. In addition to these large-scale networks, we added thalamus, hippocampus/amygdala, and brainstem[78].
