# Supplementary material for: Spatiotemporal integration of contextual and sensory information within the cortical hierarchy in human pain experience
Source: PLoS Biol. 2024 Nov 13;22(11):e3002910. doi: 10.1371/journal.pbio.3002910 (PMC11602096; doi:10.1371/journal.pbio.3002910)
Supplement: Attachment — Submitted filename: SEMIC_response_letter_first_revision_V2.pdf [file pbio.3002910.s016.pdf]

Ms. No.: PBIOLOGY-D-24-01392R1

We would like to thank the reviewers for their insightful comments and constructive suggestions and the editors of *PLOS Biology* for the opportunity to address these comments. We have included additional analysis results and more detailed descriptions to enhance the understanding of our experimental procedure and temporal mediation analysis results.

Briefly, we have

- performed additional temporal mediation analysis by varying the "free parameters" (**Fig. S8 and** *Supplementary Text*) to examine the robustness of our findings.
- included new analysis results of the simple motor task (**Fig. S5** and *Supplementary Text*) to help interpret the mediation analysis results.
- provided the temporal signal-to-noise ratio (tSNR) map (**Fig. S12**).
- added a new supplementary figure to show the actual slide presented to explain the rating scale and the pain prediction rating task (**Fig. S10**).

Overall, in this revision, we have added four new supplementary figures (**Figs. S5, S8, S10**, and **S12**).

We hope our revisions have satisfactorily addressed all the issues raised. Again, we appreciate all your insightful comments and the opportunity to improve the manuscript.

Reviewer #1: *“This manuscript presents a well-designed and executed study employing innovative techniques, such as continuous rating trajectories and temporal mediation analysis, to provide fresh insights into the neural mechanisms underlying the temporal dynamics of pain modulation. The study's presentation is excellent, and it provides with a wealth of valuable information, even in the supplementary material. With minor revisions, I believe it is suitable for publication in PLOS Biology.”*

Response: We are grateful to the reviewer for the positive evaluation of our study. We carefully addressed your comments in our point-by-point responses below.

R1-1. *“My most important remark is the apparent overabundance of "free" parameters in the temporal mediation analysis (e.g., number of components, top percentile of temporal weights, criteria for selecting brain mediators, etc). This raises concerns about the robustness of the otherwise very exciting temporal mediation results. A supplementary analysis of the robustness of the results to these parameters would be appreciated.”*

Response: We thank the reviewer for the comment. To address this concern, we conducted supplementary analyses by varying each parameter individually (as detailed below) and evaluated the spatial similarity with the brain mediators in our main findings presented in **Figs. 4-5** to ensure consistency across varying free parameters.

We examined the spatial similarity of the brain mediation maps by additionally testing:

- the number of independent components (the original parameter was 5): 15 and 30
- the threshold of temporal weights (original parameter: top 2.5%): top 1% and 5%
- the time coverage of the supra-threshold voxels (original parameter: 5%): 2.5% and 7.5%

These additional analyses aim to provide comprehensive testing for the robustness of our results and address the potential variability introduced by different hyper-parameters. As **Fig. S8** shows (see below), these results with varying hyperparameters provide patterns of brain mediation maps largely consistent with the main findings.

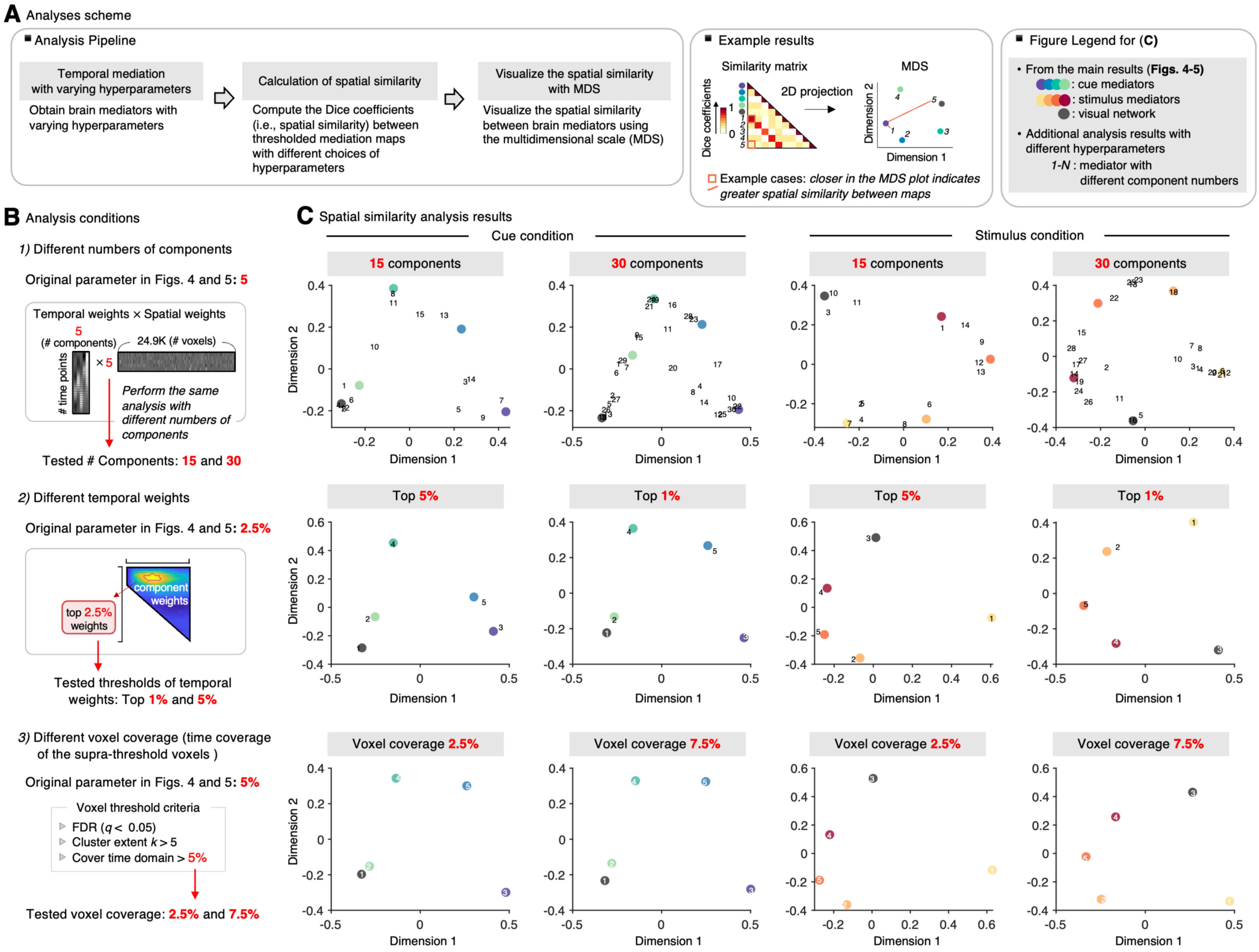
A Analyses scheme
Analysis Pipeline
Temporal mediation with varying hyperparameters
Obtain brain mediators with varying hyperparameters
Calculation of spatial similarity
Compute the Dice coefficients (i.e., spatial similarity) between thresholded mediation maps with different choices of hyperparameters
Visualize the spatial similarity with MDS
Visualize the spatial similarity between brain mediators using the multidimensional scale (MDS)
Example results
Similarity matrix
2D projection
MDS
Dice coefficients
Dimension 2
Dimension 1
Example cases: closer in the MDS plot indicates greater spatial similarity between maps
Figure Legend for (C)
From the main results (Figs. 4-5)
: cue mediators
: stimulus mediators
: visual network
Additional analysis results with different hyperparameters
1-N : mediator with different component numbers
B Analysis conditions
1) Different numbers of components
Original parameter in Figs. 4 and 5: 5
Temporal weights × Spatial weights
5 (# components)
24.9K (# voxels)
time points
× 5
Perform the same analysis with different numbers of components
Tested # Components: 15 and 30
2) Different temporal weights
Original parameter in Figs. 4 and 5: 2.5%
component weights
top 2.5% weights
Tested thresholds of temporal weights: Top 1% and 5%
3) Different voxel coverage (time coverage of the supra-threshold voxels)
Original parameter in Figs. 4 and 5: 5%
Voxel threshold criteria
FDR (q < 0.05)
Cluster extent k > 5
Cover time domain > 5%
Tested voxel coverage: 2.5% and 7.5%
C Spatial similarity analysis results
Cue condition
Stimulus condition
15 components
30 components
Top 5%
Top 1%
Voxel coverage 2.5%
Voxel coverage 7.5%
Dimension 1
Dimension 2

Fig. S8. Temporal mediation analysis with different hyperparameters. *To assess the robustness of the temporal mediation analysis results presented in* ***Figs. 4*** *and* ***5****, we performed the same analysis with different hyperparameters and evaluated the spatial similarity with the original mediation maps.* ***(A)*** *Analysis Pipeline (left): We obtained brain mediation maps using the same analysis pipeline from the original results, modifying only one hyperparameter. We then calculated the spatial similarity between thresholded maps using Dice coefficients. The spatial similarity was visualized via multidimensional scaling (MDS) in two dimensions. In the resulting MDS plots, the distance between brain mediators represents their similarity, with greater similarity indicated by closer proximity. Example results (middle): For instance, testing a new threshold for temporal weights yields five new mediation maps, one for each component. We can compute the spatial similarity among 10 mediation maps—five from the original results and five from the new analysis results. Focusing on the orange box cases, we examine the relationship between the first mediation map from the original results and the first and fifth mediation maps from the new analysis results. A shorter distance in the MDS plot reflects greater similarity between the original and first new mediation maps, while a larger distance indicates lower similarity between the original and fifth new mediation maps. Figure legend for C (right): The color scheme used in* ***Figs. 4*** *and* ***5*** *was applied to represent mediation maps for cue and stimulus intensity. The dark gray dot signifies a mediation map from the visual cortex-dominant component. The numbers in the MDS plots denote new mediation maps derived from analyses with varying hyperparameters.* ***(B)*** *We varied the number of ICA components, the percentile of temporal component weights, and the coverage of the defined temporal domain for voxel thresholding one at a time, while keeping all other analysis parameters consistent with* ***Figs. 4*** *and* ***5****.* ***(C)*** *Spatial similarity results: MDS was used to project the spatial similarities between brain mediators from the main and additional analyses into a 2-dimensional space. Colored circles represent the mediators from the original results in* ***Figs. 4*** *and* ***5****, while number markers correspond to the results from new analyses with different hyperparameters. Mediation maps with altered temporal weights and voxel coverages generally aligned with the original results. Even when more components were tested, many new brain mediation maps exhibited high similarity to the original maps. Overall, the additional analyses with varying hyperparameters produced brain mediator patterns largely consistent with the original findings.*

We have also included supplementary text to explain these additional analyses in the supplementary information section (p. 2):

Temporal mediation analysis with varying parameters

To assess the robustness of the temporal mediation analysis results presented in ***Figs. 4*** *and* ***5****, we performed a supplementary analysis with different hyperparameters and evaluated the spatial similarity with the original mediation maps. Specifically, we tested: 1) the number of ICA components = 15 and 30 (the original parameter was 5), 2) the threshold of temporal weights = 1% and 5% (original parameter = 2.5%), and 3) the time coverage of the supra-threshold voxels = 2.5% and 7.5% (original parameter = 5%), while keeping all other analysis parameters consistent with* ***Figs 4*** *and* ***5****. Then, we calculated spatial similarities between brain mediation maps, including results from our main findings in* ***Figs 4*** *and* ***5*** *and new analysis*

*results using Dice coefficients. These similarities were visualized in 2-dimensional spaces via multidimensional scaling (MDS), which represents the distance between brain mediators. See **Fig. S8** for results.*

We then discuss these additional results in the discussion section. We have added and revised the following sentences in the discussion section (p. 21-22):

*The temporal mediation analyses in our study, while informative, are not without limitations. First, hyperparameters, such as the number of independent components and thresholds for temporal domains and spatial maps, were somewhat arbitrary. To assess the robustness of our results against these arbitrary hyperparameter choices, we conducted a supplementary analysis by varying the hyperparameters and evaluated the spatial similarity with our main brain mediation maps (Supplementary Text). As shown in **Fig. S8**, the supplementary analysis results yielded brain mediation patterns largely consistent with the main findings.*

R1-2. *"In terms of the presentation, I agree that it is appealing to discuss the results in terms of a continuous Bayesian updating process. However, the study provides limited evidence that the dynamic changes in the mediation pattern are directly related to such processes. There is no direct evidence that priors are updated. If we adhere to the Bayesian theoretical framework, the results can also be explained by an increasingly precise sensory likelihood component during the continuous Bayesian integration process. (And I am not sure these results need to be forced into this framework at all.) The relevant parts in the text should be improved by acknowledging these limitations or even toned down (in the abstract, introduction, and discussion)."*

Response: We appreciate this valuable feedback. While our study was motivated by the continuous Bayesian framework, we agree that our study does not provide direct evidence linking the dynamic changes in mediation patterns to a Bayesian updating process. Accordingly, we agree that our interpretation should be presented with greater caution. We have revised the relevant sections—the abstract, introduction, and discussion—to reflect this, toning down the language to more accurately represent our findings.

We have deleted the terms "prior" and "Bayesian" and changed them to different terms in the Abstract, Introduction, and Discussion:

p. 2 (Abstract):
Pain is not a mere reflection of noxious input. Rather, it is constructed through the dynamic integration of ~~prior~~ current predictions with incoming sensory input… Our findings advance the understanding of how the brain integrates ~~prior~~ contextual and sensory information into pain experience over time.

p. 3 (Introduction):
Pain experience can be understood as a continuous integration process of incoming noxious input with ~~prior~~ contextual information, inferring the current state of both external and internal environments [1-3]. For example, the impact of expectation on pain perception, such as

placebo and nocebo effects, ~~can be conceptualized using a Bayesian framework [4, 5], in which incoming sensory information updates priors, continuously forming new pain priors [6]~~ can be understood as a dynamic process, in which the brain continuously integrates sensory inputs with contextual information [4, 5], shaping the perception by consistently forming and updating expectation (or prediction) [6].

p. 19 (Discussion):
The pain experience can be conceptualized as a continuous ~~Bayesian~~ updating process, in which predictions about the pain state are continuously updated by integrating contextual information with incoming sensory inputs.

R1-3. *"The manuscript would also benefit from improved motivation and description of the semicircular layout. The motivation should be introduced earlier in the text, rather than leaving the reader to discover in the methods section that the main reason is to address the issue of intensity rating being confounded with cursor movement distance by making the starting point equidistant from all possible ratings. Actually, I remain somewhat unconvinced that the semicircular layout does not induce bias in the ratings. As the distance from the initial point increases, the same angle might appear more extreme to the participant compared to the center line. Furthermore, if I understood correctly, participants saw their own rating history within a block, which could induce a "self-reinforcement effect" where participants strive to remain consistent with their previous ratings."*

Response: We thank the reviewer for the suggestions and insightful comments on the semicircular rating scale. Initially, we did not discuss this in the Introduction because we wanted to focus more on the study's primary research question rather than its methodological aspect. However, we fully agree that introducing our motivation earlier in the manuscript would clarify the use of this unique rating scale. See our changes below.

Also, we agree that the semicircular layout could introduce some bias. For example, as the reviewer points out, the scale has an unequal rating distance at the center versus the periphery. This means that smaller movements near the center result in larger rating changes compared to the periphery. To mitigate this, we provided extensive training and clear instructions to participants prior to the experiment. In particular, we emphasized that it was the angle, not the distance, that reflected their ratings, and that they were not required to reach the outer end of the semicircles. We added this point to the limitation in the Discussion section (see below).

Our revisions are as follows:

p. 4 (Introduction):
The application of this method faces limitations with conventional experimental paradigms that largely rely on 'button-based' or 'single value-based' behavioral assessments [9, 10, 13-15, 17], given that they are typically optimized to probe segmented processes [11]. For example, the conventional pain rating task typically asks participants to report their overall pain intensity or unpleasantness as a single value, thus treating the ongoing pain process as a single and segmented event and overlooking its inherently dynamic nature. Furthermore, in conventional

continuous rating scales in fMRI studies, rating-related motor responses are often confounded with pain intensity [18], as higher pain intensity requires further movement, e.g., to the right [19, 20]. To address these issues, we devised a semi-circular rating scale that places the starting point equidistant from all possible ratings, minimizing the effect of movement distance on ratings. This allows us to implement a 'trajectory-based' assessment.

p. 23 (Discussion):
*Lastly, the semicircular rating scale could introduce some bias as the scale has an unequal rating distance at the center versus the periphery. This leads to small initial movements near the center resulting in large changes in ratings, and large later movements in the periphery resulting in small changes in ratings. To mitigate this, we provided extensive training and clear instructions to participants prior to the experiment (see **Fig. S10** and Materials and Methods). In particular, we emphasized that it was the angle, not the distance, that reflected their ratings, and that they were not required to reach the outer end of the semicircles. However, future studies should examine the same question using different types of rating scales.*

We also want to clarify that participants did not see their own rating history. During the task, participants only observed the current position of the dot within the semicircular layout, without any visual feedback on their rating history. To clarify this, we have revised **Fig. S11** (added the information about visibility) and its caption. We also made a short video of an example trial (https://youtu.be/tR0LUDaYt38) to illustrate what participants saw in the scanner.

p. 17 of the supplementary material (**Fig. S11**):

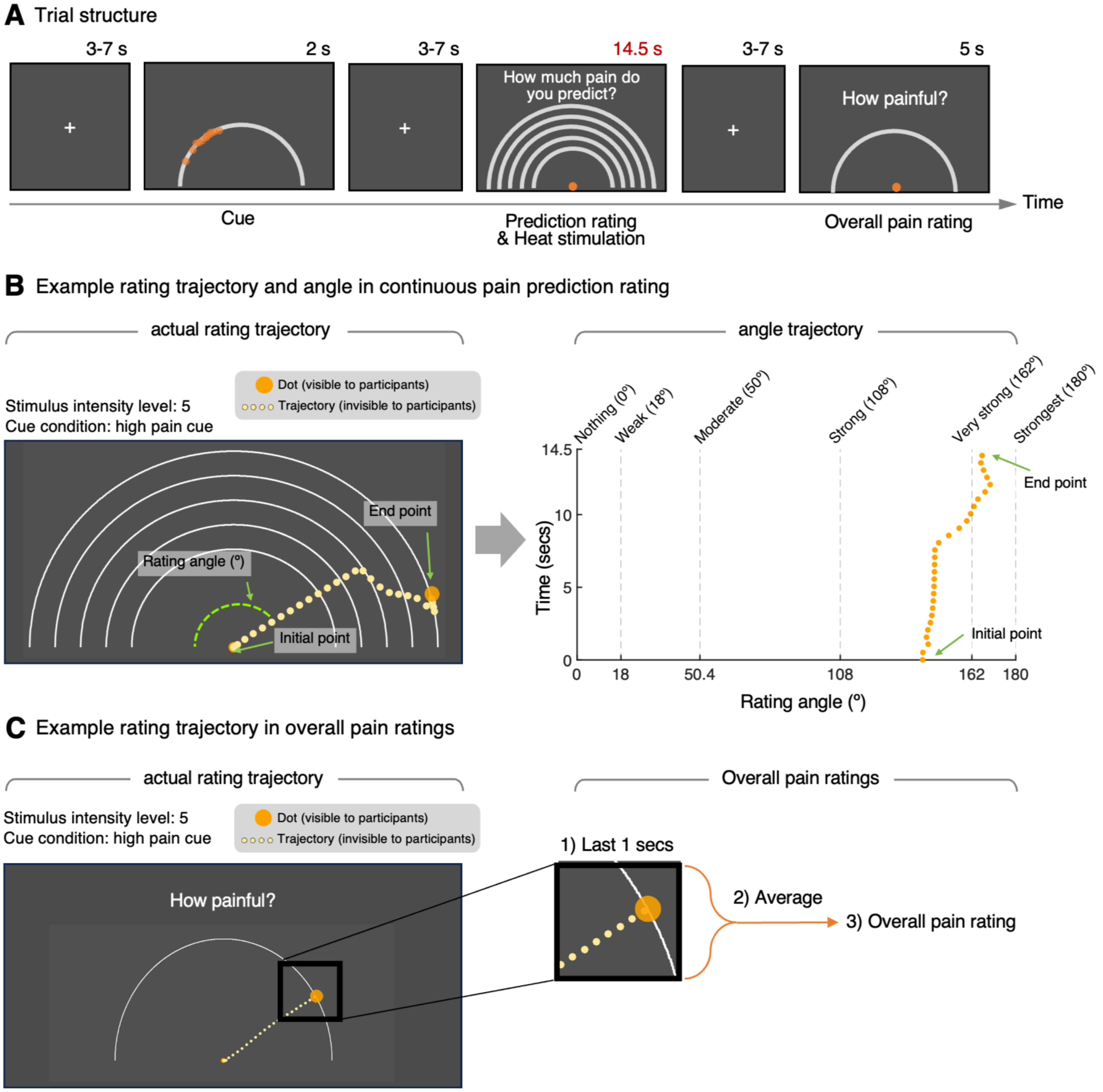

Fig. S11. Trial structure and rating trajectory examples. (A) *Schematic overview of trial structure.* ***(B)*** *Example of the continuous pain prediction rating: (Left) The figure shows an example rating trajectory over the 14.5 seconds during the pain prediction rating period. Starting from the center (i.e., the initial point), participants were asked to move the orange dot anywhere within the screen to report their continuous ratings. We emphasized that it was the angle, not the distance, that reflected participants' rating, and that they were not required to reach the outer end of the semicircles. The rating trajectory was recorded with the dot's x and y coordinates, but it was converted to the angle from the left segment of the semicircle base. (Right) The (x, y) coordinates were converted to the angles, which served as online ratings. Only the current dot location represented as the orange dot was visible to participants.* ***(C)*** *Example of the overall pain rating: (Left) The figure shows an example rating trajectory for the overall pain rating. (Right) The dot's (x, y) coordinates were converted to the angles. Then, we calculated the average of the angles of the last 1 second, which served as the overall pain rating. We made a short video of an example trial (https://youtu.be/tR0LUDaYt38) to illustrate what participants saw in the scanner.*

Minor issues

R1-4. *"A brief description of the paradigm earlier in the text would be useful. Initially, it is unclear how pain stimuli changed during the experiment. More information is needed in the introduction for readability."*

Response: We have added the following paragraph in the Introduction (p. 4-5):

> *We addressed these questions using a functional Magnetic Resonance Imaging (fMRI) experiment using a cue-induced pain modulation task. Specifically, participants were asked to continuously report their real-time prediction of pain intensity on the semi-circular rating scale using a joystick while receiving a gradually changing heat stimulus. Pain expectation was induced using pain-predictive cues based on social information, represented as dots on the rating scale before heat stimulation. The rating trajectories were operationalized as an indicator of a real-time pain integration process[11, 12, 24]. Using this trajectory dynamic information, we conducted multilevel general linear models to examine the ongoing integration process. Then, employing temporal mediation analysis combined with a data-driven approach, we identified temporal mediation profiles, which are spatiotemporal patterns of brain regions that mediate this ongoing integration process of cues and stimulus intensity over time.*

R1-5. *"Some parts of the introduction are somewhat redundant (last two paragraphs)."*

Response: We have reorganized and revised the last two paragraphs of the Introduction (p. 4-5):

> *The application of this method faces limitations with conventional experimental paradigms that largely rely on 'button-based' or 'single value-based' behavioral assessments [9, 10, 13-15, 17], given that they are typically optimized to probe segmented processes [11]. For example, the conventional pain rating task typically asks participants to report their overall pain intensity or unpleasantness as a single value, thus treating the ongoing pain process as a single and segmented event and overlooking its inherently dynamic nature. Furthermore, in conventional continuous rating scales in fMRI studies, rating-related motor responses are often confounded with pain intensity [18], as higher pain intensity requires further movement, e.g., to the right [19, 20]. To address these issues, we devised a semi-circular rating scale that places the starting point equidistant from all possible ratings, minimizing the effect of movement distance on ratings. This allows us to implement a 'trajectory-based' assessment. The 'trajectory-based' measurements have proven effective in capturing real-time temporal dynamics of motor or cognitive processes in multiple contexts [12, 21-26]. The trajectory-based rating method combined with temporal mediation analysis offers a novel avenue to scrutinize the details of a dynamic process of how the brain integrates sensory inputs with prior contextual information over time.*

> *The primary research questions of the current study are as follows. (1) Can we reveal the continuous integration process underlying pain perception with the trajectory-based measurement? and (2) Can we identify the specific timing and brain regions that mediate the temporal dynamics of this integration process? We addressed these questions using a functional Magnetic Resonance Imaging (fMRI) experiment using a cue-induced pain modulation task. Specifically, participants were asked to continuously report their real-time prediction of pain intensity on the semi-circular rating scale using a joystick while receiving a gradually changing heat stimulus. Pain expectation was induced using pain-predictive cues based on social information, represented as dots on the rating scale before heat stimulation. The rating trajectories were operationalized as an indicator of a real-time pain integration process [11, 12, 24]. Using this trajectory dynamic information, we conducted multilevel general linear models to examine the ongoing integration process. Then, we employed temporal mediation analysis to identify spatiotemporal mediation profiles over time.*
>
> *Our hypotheses primarily focus on the potential roles of the lateral prefrontal cortices in encoding contextual information [27-32]. Furthermore, we anticipated that, during this integration process, brain regions along the cortical hierarchy (or the association-sensorimotor axis) [33-36] may exhibit distinct temporal mediation profiles for cue and stimulus effects. For example, association brain regions could play an important role in mediating the effects of contextual cue information, whereas sensorimotor brain regions may primarily mediate the stimulus effects on pain. Overall, this study seeks to elucidate when and which brain regions and networks contribute to pain construction at different temporal stages.*

R1-6. *“"Are the temporal mediation findings sensible and interpretable in terms of the large-scale functional organization of the brain?" It's a matter of taste, of course, but this might be phrased too broad to be a meaningful research question.”*

Response: We acknowledge that the original research question was too broad. We have revised the research questions (p. 4):

> *The primary research questions of the current study are as follows. (1) Can we reveal the continuous integration process underlying pain perception with the trajectory-based measurement? and (2) Can we identify the specific timing and brain regions that mediate the temporal dynamics of this integration process?*

R1-7. *“A short, precise description of what mediation means statistically would benefit the reader.”*

Response: We have added the following sentences in the Introduction section (p. 3):

> *In this study, we employed temporal mediation analysis to examine the intricate interplay between pain modulatory factors, brain activities, and behavioral outcomes. The mediation analysis is a statistical method to assess whether the relationship between an independent variable (x; cue or stimulus intensity) and a dependent variable (y; ratings) is explained by a*

mediator (m; brain activity), which must significantly reduce or eliminate the direct effect of x on y when included in the model, indicating mediation [15, 16].

R1-8. *"While the temporal mediation analysis is well explained in the methods and Figure S4 is also very-very helpful, more clarity in the Results section would be beneficial."*

Response: We appreciate the reviewer's suggestion. We have added a more detailed description of the temporal mediation analysis methods in the Results section (p. 10-11):

> *Finally, to investigate the dynamic integration process of cue and stimulus information in the brain, we utilized temporal mediation analysis, analyzing the temporal dynamics of brain mediation for the cue and stimulus effects on the continuous pain prediction ratings. To this end, we performed the whole-brain multilevel mediation analysis with combinations of 45 TR (= 20.7 seconds) heat-evoked brain activity from the single-trial finite impulse response (FIR) model and 32 segmented continuous pain prediction ratings (= 14.5 seconds) (**Fig. 3A**; see Materials and Methods for details). We included a longer duration for the brain activation maps to accommodate potential hemodynamic response delays. To help interpretation of the results, we segmented the 20.7-second brain activation period into three time domains—early (0-6 s), middle (6-13.8 s), and late (13.8-20.7 s). Similarly, the 14.5-second rating period was segmented into the following time domains—cue dominant (1-6 s), transition (6-10 s), and stimulus dominant (10-14.5 s), based on the multilevel GLM results shown in **Fig. 1E**. As illustrated in **Fig. 3**, these mediation analyses yield high-dimensional outcomes, 32 $time_{rating}$ × 45 $time_{brain}$ × 24,860 voxels. To manage this complexity, we employed a data-driven approach via Independent Component Analysis (ICA [43]) and derived 5 independent components (ICs) to identify significant and interpretable spatiotemporal domains for brain mediation. Based on the top 2.5 percentile of the temporal weights, we first defined temporal domains and then identified brain mediators for each time domain based on pre-established criteria for selecting brain mediators. Specifically, we selected voxels that covered at least 5 percent of the defined time domain and survived at FDR $q < 0.05$ with a cluster size greater than 5 voxels (see **Fig. S4** for a graphical illustration).*

R1-9. *"Why does the visual cortex mediate late ratings so much? What did the participants see in the late phase? Could it be a self-reinforcing feedback from their own ratings (confirmation bias)?"*

Response: We appreciate the reviewer's comments regarding the visual cortex. To clarify, our results indicated that for the cue effects, the visual cortex served as a mediator from the early phases of brain activity (**Fig. 4C**), while it mediated stimulus intensity during the late phase (**Fig. 5C**). Combining these two mediation patterns, the visual cortex emerged as a mediator across nearly all temporal domains. In addition, as discussed in our response to **R1-3**, participants could only see the current position of the dot within the semicircular layout, without any visual feedback regarding their rating history. Therefore, the mediation of the visual cortex is likely task-related, rather than resulting from self-reinforcing feedback. This rationale was why we wrote in the original manuscript: *"For example, motor*

and visual cortex regions appeared to mediate both cue and stimulus effects, which may be related to task-related factors, such as operating a rating apparatus and looking at the rating trajectory on the screen." (p. 17)

Involvement of the visual cortex in pain processing is known to be associated with multisensory integration [1], attention [2], or task-related components [3]. Thus, the visual cortex mediating the cue and stimulus effects in the current study could be attributed to several factors. Though we cannot pinpoint the role of visual cortex in the current study, we believe that further research is required to examine the functional contributions of the visual cortex to pain perception.

R1-10. *"Do we see path a or b effects or mediation effects in any motor areas, possibly related to the continuous rating?"*

Response: Yes, similar to the visual cortex, we also speculated that the motor cortex mediating the cue and stimulus effects (during the middle phase for the cue and the middle-to-late phase for the stimulus) could be task-related, possibly attributed to the continuous rating, as we wrote that *"For example, motor and visual cortex regions appeared to mediate both cue and stimulus effects, which may be related to task-related factors, such as operating a rating apparatus and looking at the rating trajectory on the screen."* (p. 17)

Reviewer #2

"This study presents novel findings on the temporal dynamics of cue-induced pain modulation using fMRI. The methodological approaches include a sophisticated continuous two-dimensional scale to rate predictions about upcoming pain, temporal mediation analyses to investigate the integration of predictions with sensory inputs over time, and principal gradient mapping to interpret the hierarchical integration from transmodal to unimodal brain areas in cue-induced pain modulation. These innovative and rigorous methods effectively address the research questions posed.

The statistical analyses are robust and appropriately conducted, utilizing multilevel general linear models (GLM) and bootstrapping to ensure the reliability of the results. The supplementary information provided is comprehensive and supports the main findings of the study. Additionally, the data and code availability statements reflect the authors' commitment to transparency and reproducibility. I believe that the supplementary figures and methodological details are sufficient to enable replication of the study.

I commend the authors for the thoroughness, complexity, and clarity of their manuscript. The figures are elegant and visually pleasing, clearly reflecting the significant effort and dedication invested in this work. Overall, this manuscript makes a significant contribution to the field, and the comments below can be addressed with textual changes in the manuscript."

Response: We appreciate the reviewer's positive evaluation of our study and are grateful for the recognition of the novel contributions and methodological rigor our research brings to the field. We also value the insightful feedback on both the strengths and areas for improvement. Below, we provide detailed responses to each concern and comment raised, addressing them with the aim of further enhancing the clarity and impact of our manuscript.

R2-1. *"The use of a semicircular display for assessing prediction ratings seems sub-optimal due to the unequal distribution of space available for participants. This design results in smaller spaces towards the center and larger spaces towards the periphery, leading to unequal variability in rating resolution throughout the task. Additionally, the semicircular shape introduces a non-linear scaling of pain predictions, where small angular changes near the center represent different levels of intensity change compared to the same angular changes near the periphery. Although motivated by the need to set the starting point equidistant from all other points, this design choice introduces complexity and potential confusion for participants, as the use of angle might not be intuitive. These issues should be acknowledged in the limitations section."*

Response: We appreciate the constructive feedback on the semicircular rating scale used for continuous pain prediction. The design choices were intended to position the starting point equidistant from all possible ratings and allow participants to provide immediate pain ratings by moving the cursor in any direction from the start point. However, we acknowledge that this format introduced unforeseen challenges we had not fully anticipated. For instance, as the reviewer noted, the scale exhibited unequal resolution and non-linear scaling between the center and the periphery. To address this, we provided extensive training and clear instructions to participants prior to the experiment. Specifically,

we emphasized that the angle, rather than the distance, represented their ratings and that it was unnecessary to reach the outer end of the semicircles. This limitation has been noted in the Discussion section.

Our revisions are as follows:

> p. 23 (Discussion):
> *Lastly, the semicircular rating scale could introduce some bias as the scale has an unequal rating distance at the center versus the periphery. This leads to small initial movements near the center resulting in large changes in ratings, and large later movements in the periphery resulting in small changes in ratings. To mitigate this, we provided extensive training and clear instructions to participants prior to the experiment. In particular, we emphasized that it was the angle, not the distance, that reflected their ratings, and that they were not required to reach the outer end of the semicircles. However, future studies should examine the same question using different types of rating scales.*

R2-2. *“Although the semicircular scale aims to reduce the impact of movement-related confounds by using angle rather than distance, there might still be motor confounds related to how participants move the joystick. Movements towards the outer edges might require different motor control compared to movements towards the center. It would be beneficial to clarify what motion corrections were included as regressors in the GLM models to address these potential confounds. Additionally, more information on how the "simple motor task" was used in the analyses and interpretation of the results would be important to include.”*

Response: We appreciate this thoughtful comment. We also shared the concern that joystick movements could act as a potential confounding factor. To address this issue, we incorporated 24 motion parameters as nuisance regressors in the GLM models. Additionally, we excluded trials correlated with motion parameters based on variance inflation factors (VIFs), removing any trials with VIFs exceeding 2.5 from analyses. To further ensure the robustness of our findings, we increased the sample size relative to previous studies that utilized a similar experimental paradigm (i.e., cue-induced pain modulation). These efforts have been included in the manuscript:

- *(p. 30-31) We also included additional event regressors, including cue presentation and overall pain rating, and the nuisance covariates, including dummy coding regressors for each run (i.e., run intercept), linear drift across time within each run, indicator vectors for outlier time points, 24 head motion parameters, including six movement parameters (x, y, z, pitch, roll, and yaw), their mean-centered squares, their derivatives, and squared derivatives, and five principal components of cerebrospinal fluid and white matter signal.*
- *(p. 22) First, we increased the number of participants for the fMRI task to 59, compared to previous fMRI studies that included 38 participants [13], 30 participants (Study 1 of [10]), and 34 participants (Study 2 of [10]).*
- *(p. 31) As single-trial estimates could be affected by acquisition artifacts occurring during that trial (for example, sudden motion, scanner pulse artifacts, etc.), we calculated trial-by-trial*

variance inflation factors (VIFs; a measure of design-induced uncertainty due to collinearity with nuisance regressors) using the design matrix. Any trials with VIFs that exceeded 2.5 were excluded from subsequent analyses [10, 13, 16].

We also appreciate the reviewer's comment about the simple motor task. We originally included the task in the experiment to isolate the motor component of the ratings using an MR-compatible joystick, but given the already considerable complexity of the paper, we did not analyze it. However, thanks to this comment, we performed an additional analysis, which is now included in the Supplementary Information.

The analysis results (see **Fig. S5** below) revealed substantial activation of the left (ipsilateral) somatomotor regions as well as the visual cortex region during the simple motor task. These regions are also among the common brain mediators for both cue and stimulus effects, supporting our original interpretation: *"motor and visual cortex regions appeared to mediate both cue and stimulus effects, which may be related to task-related factors, such as operating a rating apparatus and looking at the rating trajectory on the screen."* (p. 17) We have now included the detailed procedure of the simple motor task and its results in Supplementary information and have integrated these findings into the revised manuscript.

p. 17 (Results):
For example, motor and visual cortex regions appeared to mediate both cue and stimulus effects, which may be related to task-related factors, such as operating a rating apparatus and looking at the rating trajectory on the screen. This interpretation was further supported by the activation patterns of the simple motor task (Fig. ***S5****; for more details on the simple motor task, please see Supplementary Text).*

p. 2 (Supplementary Information):

Simple motor task

*Participants completed a simple motor task over two runs. In the task, participants were asked to move an orange dot to specified target locations on a semi-circular rating scale using an MR-compatible joystick (**Fig. S5A**). This task, consisting of 21 trials per run, was administered prior to the first and fourth runs of the pain prediction task. Target positions were randomly selected within five intervals, each covering approximately one-fifth of the semicircle. Due to a data-saving problem, two-run data from four participants and one-run data from 15 participants were excluded. Thus, we analyzed the simple motor task data of the remaining 55 participants. We first conducted a correlation analysis between the target location and the final position of the dot (**Fig. S5B**). The results showed that participants, on average, exhibited a high level of correlation (mean $r = 0.9425$; one-sample t-test: $t(54) = 94.6784$, $SD = 0.0738$, $p = 1.0511 \times 10^{-61}$), indicating precise task performance. Then, to examine the brain activation patterns associated with the simple motor task, we conducted an fMRI single-trial analysis using the same approach as with the pain prediction task data (see **Materials and Methods**). We conducted robust regression with the averaged single-trial brain activation map for each participant [1] (see **Fig. S5C** for the results).*

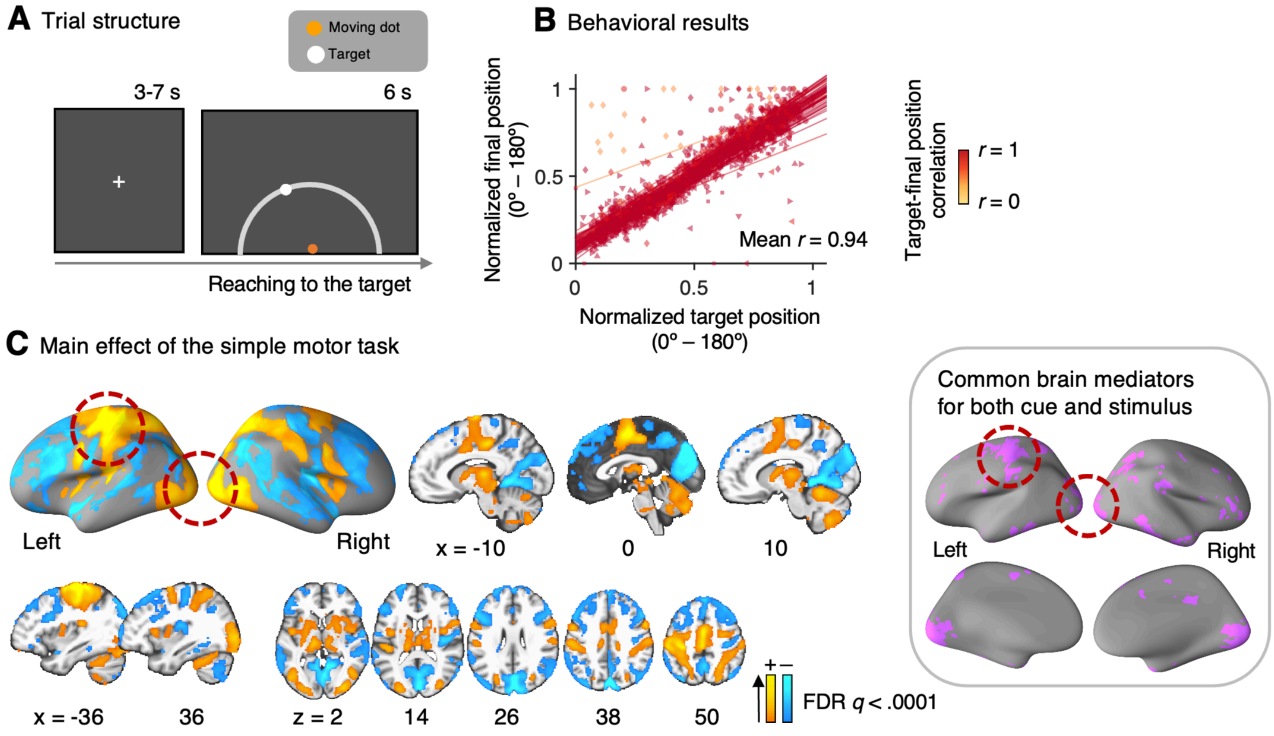

*Fig. S5. Simple motor task. (A) Participants completed a simple motor task over two runs. They were instructed to move an orange dot to specified target locations on a semi-circular rating scale using an MR-compatible joystick. Each run consisted of 21 trials, with each trial including an inter-trial interval of 3-7 seconds and 6 seconds for moving the dot. (B) Correlations between the target and actual final positions of the orange dot. Each regression line represents the relationship between the target and actual final positions for individual participants. The angle of 0 to 180° was normalized to 0-1 scale. (C) (Left) Brain maps displaying activation patterns during the simple motor task (FDR q < 0.0001 and a cluster size k < 2 voxels). (Right) Common brain mediators for both cue and stimulus intensity from **Fig. 6B**. Red dashed circles highlight the somatomotor and visual cortex regions, which served as common brain mediators for both cue and stimulus effects.*

R2-3. *"The current discussion of the transition from transmodal to unimodal brain areas does not adequately recognize the circularity of brain communications. One would expect continuous, cyclical interactions between transmodal and unimodal areas. Acknowledging and discussing this continuous interaction would enhance the manuscript's explanation of the brain's integrative processes in pain perception."*

Response: We appreciate the reviewer's comment and fully agree that continuous bidirectional interactions between transmodal (association) and unimodal (sensorimotor) regions are crucial for understanding pain perception. However, given the current experimental design and analysis approach, directly testing these interactions remains challenging, underscoring the need for future studies.

To address this comment, we have added the revised paragraph to the Discussion section (p. 21):

> *Furthermore, the temporal mediation results of our study can be interpreted within the framework of a cortical hierarchy as proposed by Mesulam [34] and further elaborated by many recent studies [33, 35, 36]. Our findings suggest a dynamic transition from association (transmodal) to sensorimotor (unimodal) regions for integrating cue and stimulus information during pain experience. This transition, from encoding expectations in the association areas to incorporating sensory inputs from sensorimotor regions, aligns with previous studies on cerebral contributions to pain perception [1, 3, 52, 53]. This does not, however, imply that the information flow or integration process is unidirectional. Instead, the continuous and reciprocal interactions between association and sensorimotor brain regions are crucial for updating current beliefs [10], adapting to new stimuli, such as through habituation, sensitization, and normalization [64], and constructing the subjective experience of pain. However, with the current experimental design and analysis approach, we could not directly test the ongoing bidirectional interactions across the cortical hierarchy, highlighting the need for future studies.*

R2-4. *“The Declaration of Helsinki should be mentioned to ensure ethical standards are addressed.”*

Response: We have added the following statement to the Materials and Methods section (p. 24):

> *The experiment was approved by the institutional review board at Sungkyunkwan University and was performed in accordance with the Declaration of Helsinki.*

R2-5. *“The phrase "write an informed consent" should be corrected to "sign the informed consent.""*

Response: We have revised the sentence as the following (p. 23-24):

> *All participants provided signed informed consent prior to the experiment in both sessions and received monetary compensation for their participation.*

Reviewer #3

"The manuscript by Gim and colleagues was a pleasure to read, and I would like to congratulate the authors on an excellent study. There are many things to like here: the manuscript is very well written, the figures adequately convey the (quite complex) sets of results in a beautiful manner, the sample size is on the large side for typical fMRI experiments, and the data are rigorously analyzed. On top of this, the authors introduce two key novel methodologies (semicircular rating scale and temporal mediation analysis) that will be highly beneficial to pain research in the future and based on which they have constructed a very elegant paradigm to look at the dynamics of integrative processes in pain perception. Overall, I strongly recommend publication of this work and only have a few points that would need to be addressed."

Response: We appreciate the reviewer's positive evaluation and kind remarks about our manuscript. We have addressed the points you raised in detail below to further enhance our manuscript.

R3-1. *"1. There are two aspects of the design that are currently unclear to me and where I believe it could be helpful if the authors were to explain the reasoning behind their choices. First, why did the thermal stimulation use such a slow ramp (5s to target temperature), considering that the chosen thermode is capable of faster ramping-behaviour to my knowledge? Second, why did they employ such a long and variable delay between cue offset and pain onset?"*

Response: Thanks for the clarification questions. First, regarding the slow ramp-up (5 seconds), given that the primary aim of the current study was to investigate the temporal dynamics of the information integration process underlying cue-induced pain modulation, we reasoned that a brief ramp-up duration (e.g., 1 or 2 seconds) would likely make it challenging to capture the relevant neural and behavioral features associated with the integration process. We have clarified this by revising the Materials and Methods section (p. 24):

> *The thermal stimulation had a duration of 12.5 seconds, comprising a 5-second ramp-up, a 5-second plateau, and a 2.5-second ramp-down (**Fig. 1A**). We opted for a slow ramp-up, as a faster ramp-up (e.g., 1 or 2 seconds) would likely hinder the ability to capture the relevant neural and behavioral features associated with the integration process.*

Second, the rationale for using long and variable intervals between the cue offset and pain onset was to improve the separation of BOLD signals related to the cue and the noxious stimulus. This approach is based on established evidence that variable inter-trial or inter-stimulus intervals provide more accurate estimates of the hemodynamic response in event-related fMRI experiments compared to fixed intervals [4]. Additionally, this variability was intended to reduce participants' ability to predict the timing of cue presentation and stimulus delivery, thereby minimizing habituation to the events. We have also clarified this by revising the Materials and Methods section (p. 28):

> *A fixation cross was displayed during the jittered inter-trial and inter-stimulus intervals, which lasted between 3 and 7 seconds, totaling 15 seconds for each trial (see **Fig. 1A**). The long and variable intervals were designed to effectively dissociate the BOLD signals associated with the*

cue and the noxious stimulus [70], and to reduce participants' ability to predict the timing of cue presentation and stimulus delivery, thereby minimizing habituation.

R3-2. *"2. While a real strength of the manuscript is the development of the semicircular rating scale (providing several advantages over a VAS), there are a few issues the authors should address. First, how do we know how participants interpret the scale, considering that it ranges from 'no sensation' to 'strongest imaginable sensation' and thus confounds innocuous temperature changes (close to the left end of the scale) with noxious ones (close to the right end of the scale)? In other words, where on the scale is the change from innocuous to painful percept occurring and will this be identical across participants? Second, how does the scale look, considering that different numbers of lines are used throughout the figures? Third, to make this novel tool useful for the pain research community, the authors should make the code available upon which the scale is based. Fourth, how do we know that participants indeed use the scale as intended, i.e. making pain 'predictions' instead of reporting pain 'intensity' (especially in later parts of the trial)?"*

Response: We appreciate the reviewer's valuable comments on our semicircular rating scale, which have helped us clarify key aspects of its design and implementation. The Figs. R1 and R2 below have been added to the revised Supplementary Information as **Fig. S10.**

First, participants were thoroughly instructed on how to use the scale before proceeding to the pain calibration task on Day 1. For this, we used a structured instruction script, and the instructions focused primarily on the anchors and corresponding labels of the scale. As noted in the original manuscript, we employed a modified version of the generalized Labeled Magnitude Scale (gLMS [5]) to define the anchors. These anchors included no sensation (0°), weak (18°), moderate (50°), strong (108°), very strong (162°), and strongest imaginable sensation (180°; see **Fig. 1D**, **Fig S10,** and **Fig. R1** below). Importantly, we emphasized that the label "Weak" marked the point where the sensation began to feel painful. To ensure participants fully understood the instructions, they were asked to explain the scale back to experimenters. **Fig. R1** shows the slide presented for this "explaining-back" procedure. If a participant was unable to explain the scale correctly, we provided the instructions again until they fully grasped the scale. Recognizing that the semicircular rating scale with gLMS labels may not be intuitively easy to use, we invested significant effort in ensuring all participants shared a consistent understanding of the scale.

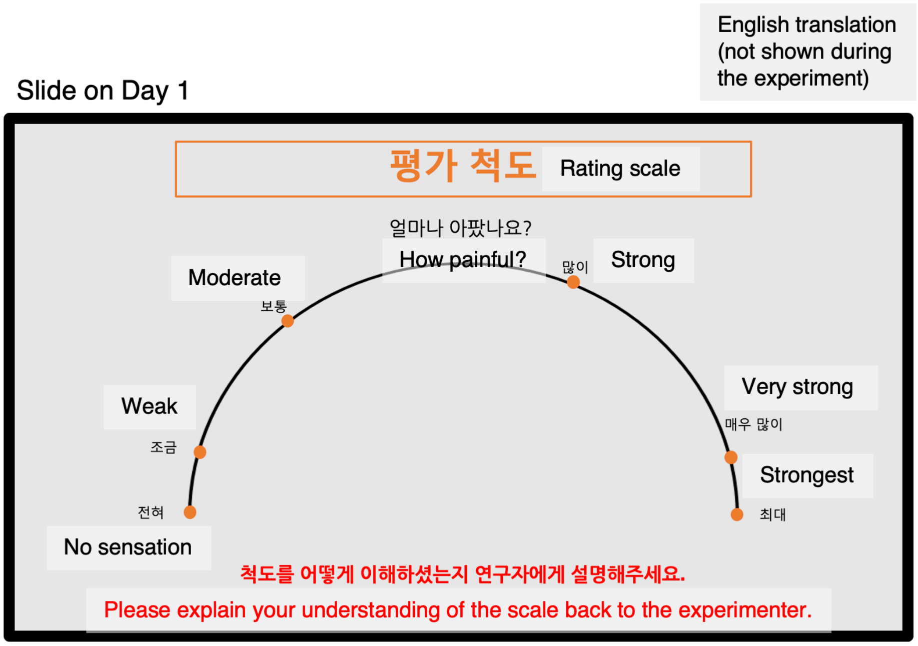

Fig. R1. The actual slide presented on Day 1 to ensure participants fully understood the scale. The English translation of the text was not displayed during the experiment.

Second, to help readers understand the semicircular rating scale, we have created a video (https://youtu.be/tR0LUDaYt38) with a sample trial showing the actual screen participants viewed in the scanner during the experiment. The link has been included in the legend of **Fig S11**.

Third, to facilitate wider use of the semicircular ratings, we have made the code available through the Figshare repository (https://figshare.com/s/9e2acec443e66571c719). We hope that this will help other researchers to implement and adapt the scale for their studies.

Fourth, on Day 2, participants were thoroughly instructed on the difference between intensity rating and prediction ratings prior to the experiment. **Fig. R2** shows the actual slides used during this procedure. As each trial included two types of ratings—continuous pain prediction ratings and overall pain intensity ratings—it was crucial for participants to fully understand this difference. Since participants had already learned the intensity rating on Day 1, the focus of the Day 2 instruction was primarily on the prediction rating and how it differed from the intensity rating. The key aspect of the prediction rating was for participants to continuously report their ongoing estimate of how painful the stimulus would be, indicating their ‘current’ prediction of the pain level. Similar to Day 1, we also asked participants to explain the pain prediction rating back to the experimenter, and if their understanding was incomplete, the procedure was repeated until they fully grasped it.

If participants understood the pain prediction ratings correctly, the ratings during the early part of each trial should have been aligned with the level of cues. Later in the trial, the ratings would then shift towards the actual level of stimulus intensity, which was confirmed by the data (**Fig. 1D** and **Fig. S2**), indicating that participants accurately distinguished between pain prediction and pain intensity ratings.

Slides on Day 2

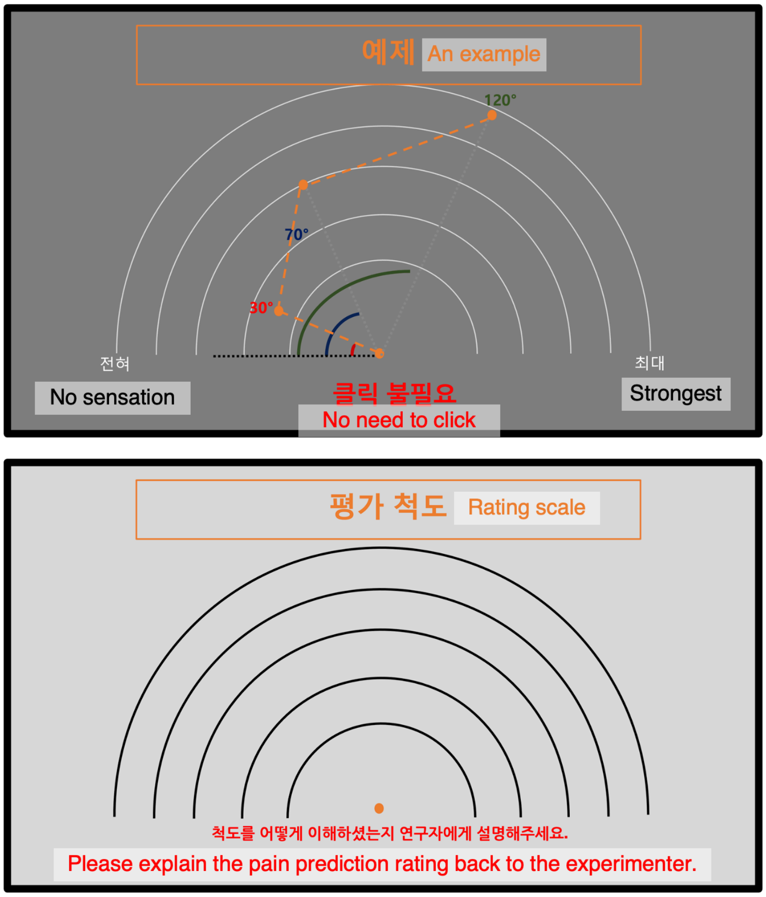

Fig. R2. The actual slides presented on Day 2 to ensure participants fully understood the prediction rating. The key aspect of the prediction rating was for participants to continuously report their ongoing estimate of how painful the stimulus would be, indicating their ‘current’ prediction of the pain level. (top) We showed participants an example of the continuous pain prediction ratings to explain the pain prediction rating and how to report it. We instructed them the angle from the start point will be regarded as a current pain prediction. We also instructed that they did not need to click the button to report the prediction continuously, highlighting the difference between the prediction ratings and the intensity ratings. (bottom) We asked participants to explain what they understood about the pain prediction back to the experimenter. If their understanding was incomplete, the procedure was repeated until they fully grasped it. The English translation of the text was not displayed during the actual experiment.

We have also added brief explanations on **Fig S10** in the Materials and Methods section (p. 25):

> *The entire experiment unfolded over two-day sessions. In the first day session, we executed the pain calibration task to induce similar levels of pain experience across participants [8, 15]. During the second day session, we conducted the fMRI pain experiment. At the beginning of each session, we provided an overview including the session's duration, the tasks, and the use of the scale. After the overview, we ensured participants understood how to use the rating*

*scale and the upcoming tasks by asking them to explain the scale and the task procedures (see **Fig. S10** for more information). If participants were unable to accurately describe these elements, we provided further explanation until they demonstrated a clear understanding. This aimed to ensure consistent and accurate use of the scale throughout the experiment.*

R3-3. *"3. There are several analysis choices where I am not able to understand the authors reasoning / approach. First, I am not able to follow why a different coding of predictors was used in the GLMs for overall pain ratings and continuous pain ratings (especially considering that cue weighting order was changed); this is even more relevant, considering that the authors go back to their original coding scheme when using the ROI-mediation analysis"*

Response: We appreciate the reviewer's comment on this issue. As noted, we employed two different coding schemes for the predictors in the GLMs, but only the multilevel GLMs for continuous prediction ratings used a different coding scheme (**Fig. 1E**). Specifically, the cue levels were coded as 0.22, 0, and 0.77 for low, no, and high pain cues, respectively, and the stimulus levels as 0.3, 0.4, 0.5, 0.6, and 0.7. In contrast, all other analyses used a coding scheme of -1, 0, 1 for low, no, and high pain cues, and 1, 2, 3, 4, and 5 for the stimulus levels.

The reason that we used the different coding schemes in the multilevel GLMs for continuous prediction ratings was to ensure comparability between regressors by aligning the scale of beta coefficients (i.e., effect magnitudes). While the other analyses did not require direct comparisons between cue and stimulus effects, the results shown in **Fig. 1E** required direct comparisons to define cue-dominant, transition, and stimulus-dominant time domains, as illustrated in **Fig. 3B**.

The coding scheme for the multilevel GLMs was based on the mean ratings of cue and stimulus levels on a normalized rating scale ranging from 0 to 1. For example, 0.22 and 0.77 represented the mean rating positions for low and high pain cues, respectively. We refer to this as "scale coding." Using this scheme allows us to capture cue and stimulus effects in the same unit. In this way, the beta coefficients' units become interpretable, enabling us to compare the effect magnitudes between cue and stimuli.

While the comparability and interpretability were affected by the coding scheme, this had no impact on the statistical outcomes. As shown in **Fig. R3**, while the coding schemes influenced the magnitudes of beta coefficients (**Fig. R3A**), the correlation between them was nearly perfect (*r*s > 0.99; **Fig. R3B**), indicating that the statistical results were unaffected.

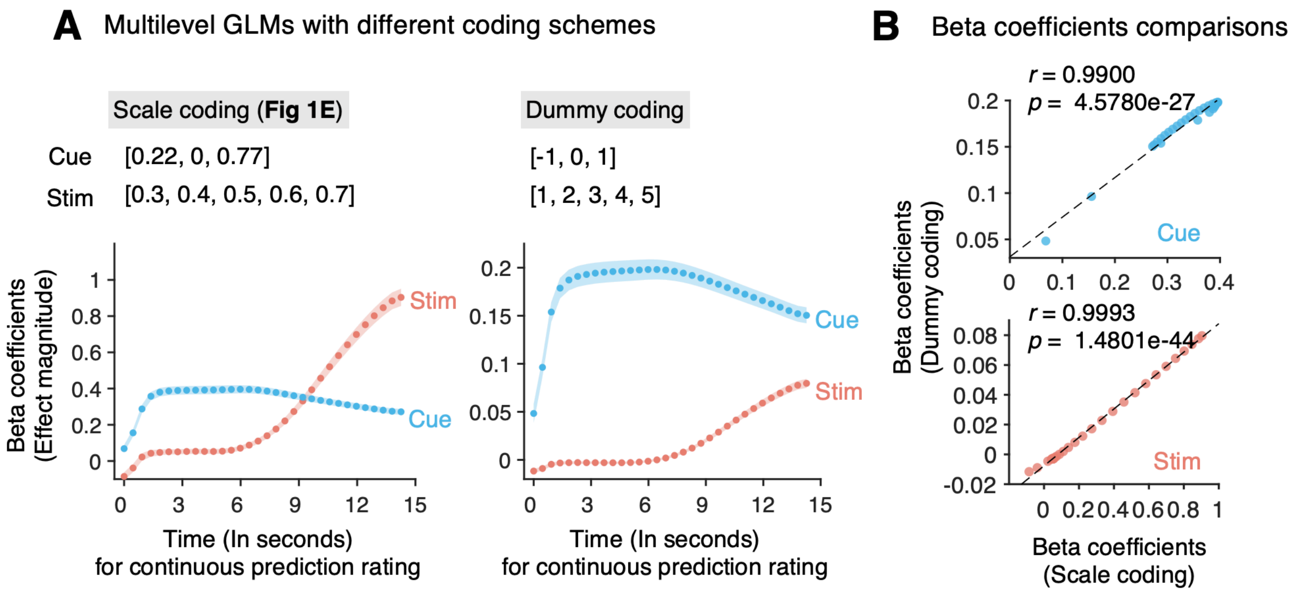

Fig. R3. Comparison between scale and dummy coding for multilevel GLM results of continuous pain prediction ratings. We compared the multilevel GLM results for continuous pain prediction ratings between the scale and dummy coding schemes. The results presented in **Fig. 1E** were based on the scale coding. **(A)** Temporal patterns of beta coefficients (i.e., effect magnitudes) over time were similar across the two coding schemes, although the magnitude of beta coefficients differed. Shading represents within-subject standard error of the mean (s.e.m.). **(B)** The second-level beta coefficients between the coding schemes were almost perfectly correlated, $r = 0.9900$, $p = 4.5780e\text{-}27$ for cue, $r = 0.9993$, $p = 1.4801e\text{-}44$ for stimulus. Blue represents cue effects, and red represents stimulus effects.

To clarify this, we have revised and included a more detailed description, along with additional analysis results, in the Materials and Methods section (p. 30):

*In this analysis, to help comparisons of the effect magnitudes between the cue and stimulus intensity effects, we coded the cue conditions and stimulus intensity levels based on the normalized rating scale (i.e., 0-1 for 0°-180°). More specifically, the low pain cue, high pain cue, and no cue conditions were coded as 0.22 (39.6°), 0,77(138.6°), and 0, respectively, and the stimulus intensity levels 1 to 5 were coded as 0.3 (54°), 0.4 (72°), 0.5 (90°), 0.6 (108°), and 0.7 (126°), respectively. These coding corresponds to the positions of the stimulus intensity levels and the mean positions of the cues on the semi-circular rating scale (**Fig. 1B**). This coding scheme allows us to capture cue and stimulus effects in the same unit, making the beta coefficients comparable. For significance testing, we conducted bootstrap tests with 10,000 iterations and corrected the results for multiple comparisons using the Bonferroni correction. In addition, we compared the current results (**Fig. 1E**) with those obtained using a different coding scheme of -1, 0, 1 for low, no, and high pain cues, and 1, 2, 3, 4, and 5 for the stimulus levels. Although the absolute magnitudes of the beta coefficients were influenced by the coding schemes, the correlation between the beta coefficients was nearly perfect (rs > 0.99), indicating that the use of different coding schemes did not affect the statistical outcomes.*

R3-4. *“Second, I am not able to understand how the design matrix looked for their fMRI single trial analysis: did they have one large design matrix where each trial was modelled separately or did they construct several design matrices (one for each trial, with the remaining trials in one regressor)?”*

Response: The former is correct. We modeled fMRI single-trial analysis with a single design matrix by including each trial’s regressor separately, which is consistent with the “beta series” approach [6].

We have added this explanation in the Methods section (p. 30-31):

> *In this analysis, we modeled the heat stimulation epoch of each trial with a boxcar function regressor, convolved with a canonical hemodynamic response function, and included the regressors for all trials in a single design matrix. We also included additional event regressors, including cue presentation and overall pain rating, and the nuisance covariates, including dummy coding regressors for each run (i.e., run intercept), linear drift across time within each run, indicator vectors for outlier time points, 24 head motion parameters, including six movement parameters (x, y, z, pitch, roll, and yaw), their mean-centered squares, their derivatives, and squared derivatives, and five principal components of cerebrospinal fluid and white matter signal.*

R3-5. *“Third, to my knowledge in SPM's FIR approach there is no convolution with the HRF - why did the authors use such a combination of basis functions?”*

Response: Given that the temporal dynamics of cue-induced pain modulation was the main target of the current study, we wanted to develop a method to analyze brain activity at a TR-by-TR resolution. By utilizing the FIR approach, we were able to analyze TR-by-TR brain activity. We also used the HRF-convolved single-trial model, but only to validate our main findings, such as the whole-brain mediation presented in **Fig. 2**.

To avoid confusion, we have revised the following in the *Materials and Methods* section:

> p. 31:
> *The single-trial brain activation maps derived from this analysis were included as mediators in whole-brain multilevel mediation analysis (**Fig. 2**).*
>
> p. 31:
> *TR-level brain activation maps were included as a mediator in the multilevel temporal mediation analysis (**Fig. 3A**).*

R3-6. *“Fourth, I cannot follow when, how and why they used temporal smoothing during the temporal mediation analysis - was this for regularization purposes and if so, on which data was it performed (fMRI, ratings, both)?”*

Response: We apologize for unclear description on this. First, for the continuous pain ratings, we performed temporal smoothing during preprocessing, and there was no additional temporal smoothing during the temporal mediation analysis. It was described in the "Continuous rating data preprocessing" section in Materials and Methods (p. 29): *"we resampled the ratings to 50 Hz using linear interpolations [24], yielding 726 data points for the pain prediction ratings, and applied the Gaussian smoothing (window size = 71) to filter out the irregularities such as noise or jitter of the rating trajectory."*

Second, for the TR-level single-trial brain activation maps, we applied temporal smoothing to a total of 45 TRs for each trial for the temporal mediation analysis to reduce TR-level noise.

We have revised the following sentence in the *Materials and Methods* section (p. 32):

> *Brain data were resampled to a resolution of 4 mm^3 using FLIRT in FSL to reduce computational load, resulting in 24,860 voxels for the whole brain. We subsequently applied temporal smoothing to the resampled brain data using a Gaussian kernel with a window size of 5 via the smoothdata.m function in MATLAB (MathWorks), to reduce noise at the TR level.*

R3-7. *"Most importantly, why was the ICA limited to five components - were other dimensionalities explored (if so, what were the outcomes; if not, why focus on exactly five components)? While it is nice to see that the authors mention this limitation in the Discussion, I believe it is important to address this analytically (if feasible), considering that it strongly shapes the outcome of the temporal mediation."*

Response: We appreciate the reviewer's comment. A similar comment was made by the reviewer #1 (see **R1-1**), and to address the concerns, we conducted supplementary analyses by varying free parameters including the number of independent components. We then evaluated the spatial similarity with the brain mediators in our main findings presented in **Figs. 4-5** to ensure consistency across varying free parameters. For the number of components, we additionally tested 15 and 30.
As **Fig. S8** shows (see below), the additional analysis results with varying hyperparameters provide patterns of brain mediation maps largely consistent with the main findings.

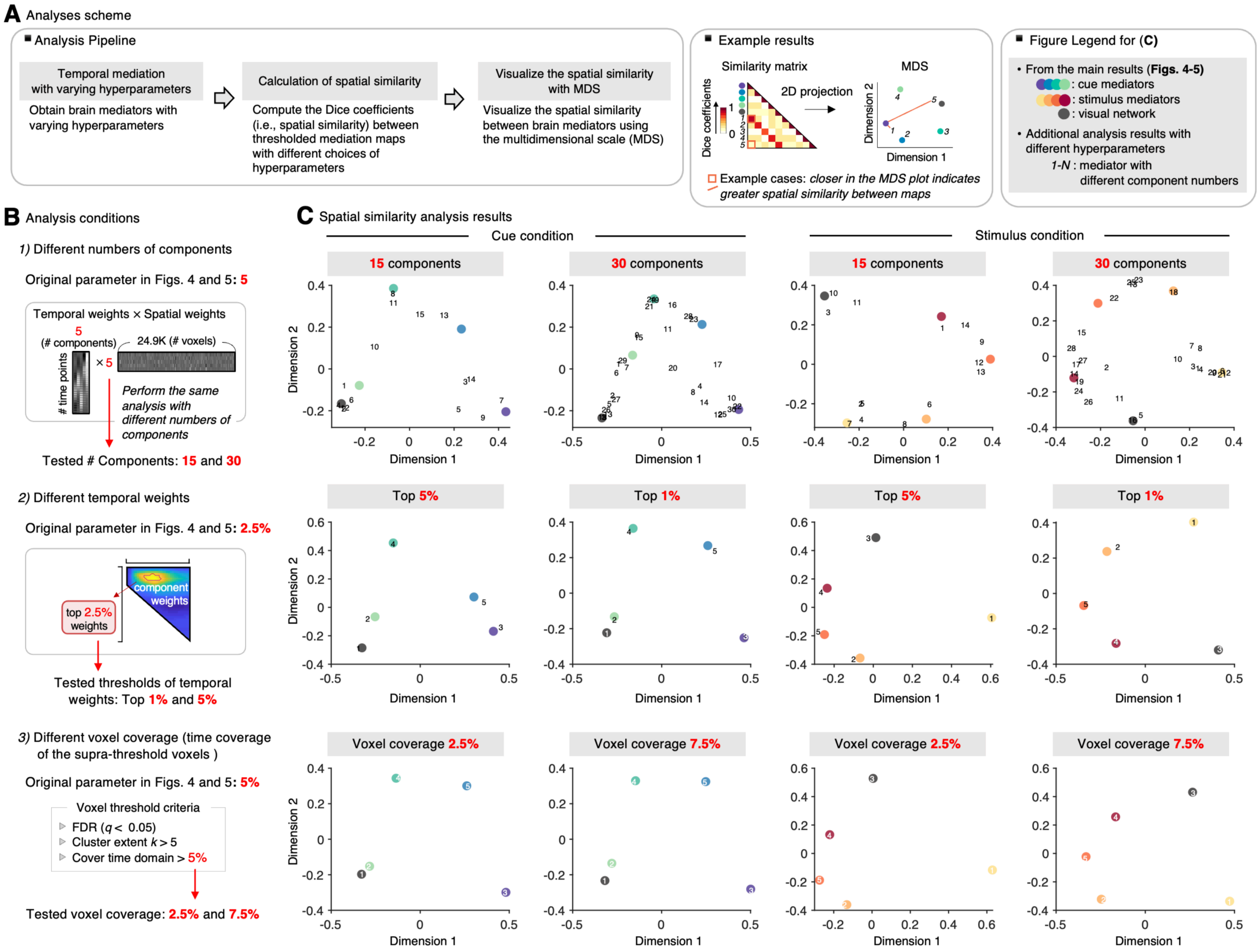
A Analyses scheme
Analysis Pipeline
Temporal mediation with varying hyperparameters
Obtain brain mediators with varying hyperparameters
Calculation of spatial similarity
Compute the Dice coefficients (i.e., spatial similarity) between thresholded mediation maps with different choices of hyperparameters
Visualize the spatial similarity with MDS
Visualize the spatial similarity between brain mediators using the multidimensional scale (MDS)
Example results
Similarity matrix
2D projection
MDS
Dice coefficients
Dimension 1
Dimension 2
Example cases: closer in the MDS plot indicates greater spatial similarity between maps
Figure Legend for (C)
From the main results (Figs. 4-5)
: cue mediators
: stimulus mediators
: visual network
Additional analysis results with different hyperparameters
1-N : mediator with different component numbers
B Analysis conditions
1) Different numbers of components
Original parameter in Figs. 4 and 5: 5
Temporal weights × Spatial weights
5 (# components)
24.9K (# voxels)
time points
× 5
Perform the same analysis with different numbers of components
Tested # Components: 15 and 30
2) Different temporal weights
Original parameter in Figs. 4 and 5: 2.5%
component weights
top 2.5% weights
Tested thresholds of temporal weights: Top 1% and 5%
3) Different voxel coverage (time coverage of the supra-threshold voxels)
Original parameter in Figs. 4 and 5: 5%
Voxel threshold criteria
FDR (q < 0.05)
Cluster extent k > 5
Cover time domain > 5%
Tested voxel coverage: 2.5% and 7.5%
C Spatial similarity analysis results
Cue condition
Stimulus condition
15 components
30 components
Top 5%
Top 1%
Voxel coverage 2.5%
Voxel coverage 7.5%
Dimension 1
Dimension 2

Fig. S8. Temporal mediation analysis with different hyperparameters. *To assess the robustness of the temporal mediation analysis results presented in* ***Figs. 4*** *and* ***5****, we performed the same analysis with different hyperparameters and evaluated the spatial similarity with the original mediation maps.* ***(A)*** *Analysis Pipeline (left): We obtained brain mediation maps using the same analysis pipeline from the original results, modifying only one hyperparameter. We then calculated the spatial similarity between thresholded maps using Dice coefficients. The spatial similarity was visualized via multidimensional scaling (MDS) in two dimensions. In the resulting MDS plots, the distance between brain mediators represents their similarity, with greater similarity indicated by closer proximity. Example results (middle): For instance, testing a new threshold for temporal weights yields five new mediation maps, one for each component. We can compute the spatial similarity among 10 mediation maps—five from the original results and five from the new analysis results. Focusing on the orange box cases, we examine the relationship between the first mediation map from the original results and the first and fifth mediation maps from the new analysis results. A shorter distance in the MDS plot reflects greater similarity between the original and first new mediation maps, while a larger distance indicates lower similarity between the original and fifth new mediation maps. Figure legend for C (right): The color scheme used in* ***Figs. 4*** *and* ***5*** *was applied to represent mediation maps for cue and stimulus intensity. The dark gray dot signifies a mediation map from the visual cortex-dominant component. The numbers in the MDS plots denote new mediation maps derived from analyses with varying hyperparameters.* ***(B)*** *We varied the number of ICA components, the percentile of temporal component weights, and the coverage of the defined temporal domain for voxel thresholding one at a time, while keeping all other analysis parameters consistent with* ***Figs. 4*** *and* ***5****.* ***(C)*** *Spatial similarity results: MDS was used to project the spatial similarities between brain mediators from the main and additional analyses into a 2-dimensional space. Colored circles represent the mediators from the original results in* ***Figs. 4*** *and* ***5****, while number markers correspond to the results from new analyses with different hyperparameters. Mediation maps with altered temporal weights and voxel coverages generally aligned with the original results. Even when more components were tested, many new brain mediation maps exhibited high similarity to the original maps. Overall, the additional analyses with varying hyperparameters produced brain mediator patterns largely consistent with the original findings.*

We have also included supplementary text to explain these additional analyses in the supplementary information section (p. 2):

Temporal mediation analysis with varying parameters

To assess the robustness of the temporal mediation analysis results presented in ***Figs. 4*** *and* ***5****, we performed a supplementary analysis with different hyperparameters and evaluated the spatial similarity with the original mediation maps. Specifically, we tested: 1) the number of ICA components = 15 and 30 (the original parameter was 5), 2) the threshold of temporal weights = 1% and 5% (original parameter = 2.5%), and 3) the time coverage of the supra-threshold voxels = 2.5% and 7.5% (original parameter = 5%), while keeping all other analysis parameters consistent with* ***Figs 4*** *and* ***5****. Then, we calculated spatial similarities between brain mediation maps, including results from our main findings in* ***Figs 4*** *and* ***5*** *and new analysis*

> *results using Dice coefficients. These similarities were visualized in 2-dimensional spaces via multidimensional scaling (MDS), which represents the distance between brain mediators. See **Fig. S8** for results.*

We then discuss these additional results in the discussion section. We have added and revised the following sentences in the discussion section (p. 21-22):

> *The temporal mediation analyses in our study, while informative, are not without limitations. First, hyperparameters, such as the number of independent components and thresholds for temporal domains and spatial maps, were somewhat arbitrary. To assess the robustness of our results against these arbitrary hyperparameter choices, we conducted a supplementary analysis by varying the hyperparameters and evaluated the spatial similarity with our main brain mediation maps (Supplementary Text). As shown in **Fig. S8**, the supplementary analysis results yielded brain mediation patterns largely consistent with the main findings.*

Minor issues

R3-8. *"Based upon which reasoning did the authors decide on a sample size of 84 (completing first session) or 59 (completing entire experiment)?"*

Response:
We originally aimed for a final sample size of 60 participants and therefore oversampled to reach this target. Why 60, then? Because there was no prior study that conducted the same temporal mediation analysis, the power analysis was not possible. Thus, we used the previous fMRI studies that utilized a similar experimental paradigm (i.e., cue-induced pain modulation) as references and tried to increase the sample size, as we stated in the manuscript, *"we increased the number of participants for the fMRI task to 59, compared to previous fMRI studies that included 38 participants [13], 30 participants (Study 1 of [10]), and 34 participants (Study 2 of [10])."* (p. 22)

R3-9. *"The second paragraph describing the 'Pain calibration task' is very hard to follow - I would suggest the authors try to make their approach clearer here. It would also be helpful if the authors could explain in more detail how the 'pain prediction task with eight runs' worked during the structural data acquisition."*

Response: First, we have revised the second paragraph of the "Day 1: Pain calibration task" section to improve readability (p. 25-26):

> *In the pain calibration task, participants reported pain intensity ratings for twelve heat stimulations. Initially, three predetermined heat temperatures (43.4, 45.4, and 47.4°C) were delivered in random order. Based on the pain ratings for these stimuli, we fitted a linear regression model to predict pain ratings (dependent variable) using temperature levels (independent variable). For subsequent trials, the temperature levels were determined using this model [8, 15]. Specifically, we estimated three temperatures corresponding to low,*

> *medium, and high stimulus intensity levels based on the linear regression model. Low, medium, and high stimulus intensity levels were operationalized as 30% (i.e., 54°), 50% (90°), and 70% (126°) of the semi-circle rating scale. One of these three stimulus intensity levels was delivered for the next trial in a pseudo-random order, ensuring no consecutive repetition of the same stimulus intensity. This procedure was repeated for each trial up to the 12th trials—i.e., for each N-th trial, the temperature was determined by fitting a new linear regression model using the temperature intensities and corresponding pain ratings from the first to the (N-1)-th trials. Finally, we determined five temperature levels for each participant, corresponding to five stimulus intensity levels (30, 40, 50, 60, and 70% of the scale, equating 54°, 72°, 90°, 108°, and 126°, respectively; see **Fig. 1B**). These five temperatures, derived from the final linear regression model of each participant, were used in the fMRI pain prediction task.*

Second, we apologize for the confusion caused by a typo (p. 26). "Eight *runs"* should be "eight *trials."* During the structural scan, participants completed eight trials of the pain prediction task, not runs. We have corrected the error.

R3-10. *"Why were the two motor task runs acquired (I did not see any results based on these runs, though they could be informative for example with respect to sensorimotor cortex activation considering the ongoing rating)?"*

Response: We appreciate the reviewer's comment regarding the simple motor task. We originally included the task in the experiment to isolate the motor component of the ratings using an MR-compatible joystick, but given the already considerable complexity of the paper, we did not analyze it. However, thanks to this comment (and **R2-2**), we performed an additional analysis, which is now included in the Supplementary Information.

The analysis results (see **Fig. S5** below) revealed substantial activation of the left (ipsilateral) somatomotor regions as well as the visual cortex region during the simple motor task. These regions are also among the common brain mediators for both cue and stimulus effects, supporting our original interpretation: *"motor and visual cortex regions appeared to mediate both cue and stimulus effects, which may be related to task-related factors, such as operating a rating apparatus and looking at the rating trajectory on the screen."* (p. 17) We have now included the detailed procedure of the simple motor task and its results in Supplementary information and have integrated these findings into the revised manuscript.

> p. 17 (Results):
> *For example, motor and visual cortex regions appeared to mediate both cue and stimulus effects, which may be related to task-related factors, such as operating a rating apparatus and looking at the rating trajectory on the screen. This interpretation was further supported by the activation patterns of the simple motor task (**Fig. S5**; for more details on the simple motor task, please see Supplementary Text).*

> p. 2 (Supplementary Information):

Simple motor task

*Participants completed a simple motor task over two runs. In the task, participants were asked to move an orange dot to specified target locations on a semi-circular rating scale using an MR-compatible joystick (**Fig. S5A**). This task, consisting of 21 trials per run, was administered prior to the first and fourth runs of the pain prediction task. Target positions were randomly selected within five intervals, each covering approximately one-fifth of the semicircle. Due to a data-saving problem, two-run data from four participants and one-run data from 15 participants were excluded. Thus, we analyzed the simple motor task data of the remaining 55 participants. We first conducted a correlation analysis between the target location and the final position of the dot (**Fig. S5B**). The results showed that participants, on average, exhibited a high level of correlation (mean r = 0.9425; one-sample t-test: $t(54) = 94.6784$, SD = 0.0738, $p = 1.0511\times10^{-61}$), indicating precise task performance. Then, to examine the brain activation patterns associated with the simple motor task, we conducted an fMRI single-trial analysis using the same approach as with the pain prediction task data (see **Materials and Methods**). We conducted robust regression with the averaged single-trial brain activation map for each participant [1] (see **Fig. S5C** for the results).*

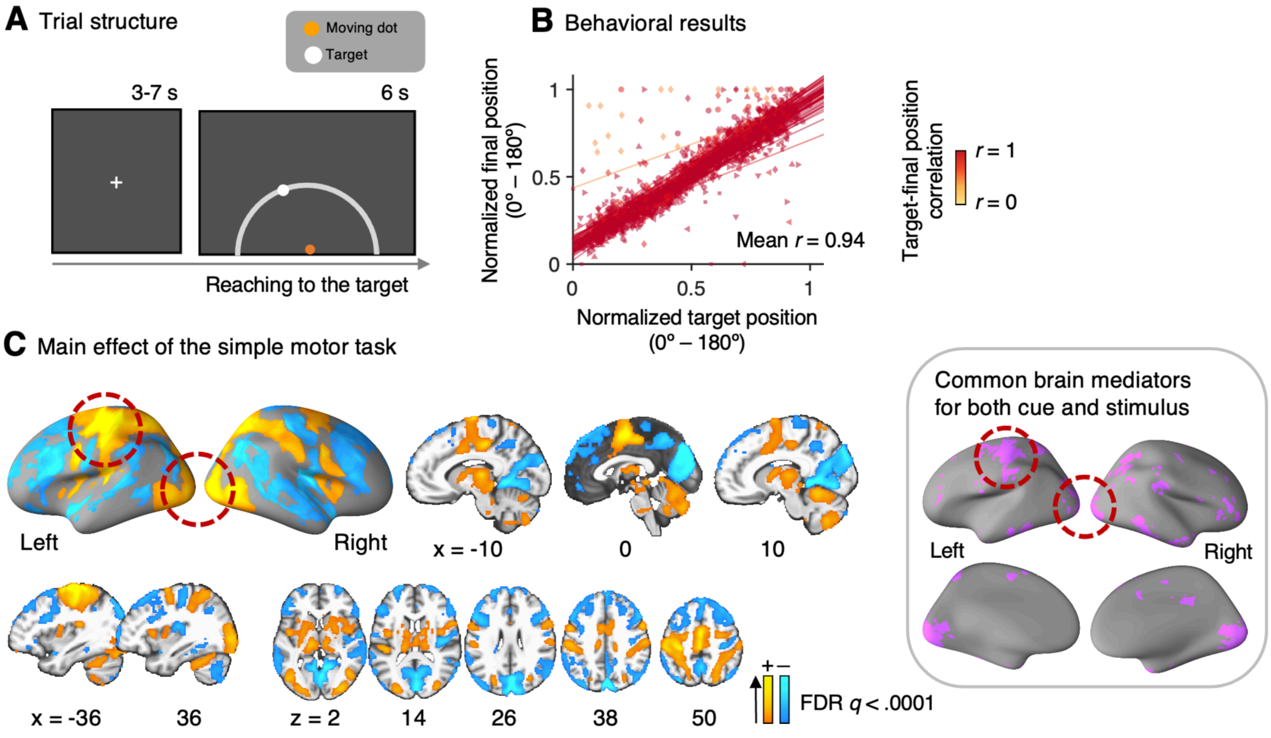

***Fig. S5. Simple motor task. (A)** Participants completed a simple motor task over two runs. They were instructed to move an orange dot to specified target locations on a semi-circular rating scale using an MR-compatible joystick. Each run consisted of 21 trials, with each trial including an inter-trial interval of 3-7 seconds and 6 seconds for moving the dot. **(B)** Correlations between the target and actual final positions of the orange dot. Each regression line represents the relationship between the target and actual final positions for individual participants. The angle of 0 to 180° was normalized to 0-1 scale. **(C)** (Left) Brain maps displaying activation patterns during the simple motor task (FDR q < 0.0001 and a cluster size*

*k < 2 voxels). (Right) Common brain mediators for both cue and stimulus intensity from **Fig. 6B**. Red dashed circles highlight the somatomotor and visual cortex regions, which served as common brain mediators for both cue and stimulus effects.*

R3-11. *"What was the purpose of the 'other-pain question' and where do the authors report the data from this question?"*

Response: The purpose of including the 'other-pain question' was to address a separate research question that is not covered in the current manuscript. Briefly, the inclusion of the 'other-pain question' aimed to investigate the brain mechanisms underlying perspective-taking in pain. Additionally, it could reduce the motor preparation component during the interval before pain rating. In case we analyze the delay period between heat stimulation and the overall pain rating, we considered it beneficial to include the 'other-pain question.'

We have detailed this in the Method section (p. 27-28):

> *Within a run of 18 trials, the self-pain question was asked for 11 trials, and the other-pain question for 7 trials. The other-pain question was included to address a separate research question that is not covered in the current manuscript. The 11 trials with the self-pain question included all combinations of stimulus intensity levels and cue conditions, allowing us to analyze the cue effects on the overall pain ratings (**Figs. 1C** and **2**).*

R3-12. *"A multi-band factor of 8 is quite aggressive - did the authors consider possible slice-leakage artefacts? Also, was there no in-plane acceleration via GRAPPA (if not, how strong were distortions of the EPI data) and no partial-Fourier employed? Considering their very fast TR and low flip-angle, could they report tSNR measures in a supplementary figure?"*

Response: We employed a multi-band factor of 8 to implement the shortest TR to effectively capture the temporal dynamics of brain activity. Although GRAPPA and/or (phase) partial-Fourier were not utilized, we applied spatial smoothing to reduce noise and artifacts, ensuring the reliability of our data.

We have revised the following paragraph to add more detailed information on fMRI acquisition in the Materials and Method section (p. 28):

> *We acquired functional images using the T2*-weighted echo-planar image (EPI) protocol (TR = 460 ms; TE = 27.20 ms; multi-band factor = 8; field of view = 220 mm; flip angle = 44°; the number of interleaved slices = 56; voxel size = 2.7 mm^3; pre-scan normalize; iPAT = none; anterior-to-posterior phase encoding direction). For all functional scans, we discarded the initial 18 TR images to allow for image intensity stabilization (see **Fig. S12** for the temporal signal-to-noise ratio map). Prior to statistical analyses, we implemented the following preprocessing steps: outlier detection, realignment, distortion correction, coregistration and normalization, and spatial smoothing.*

We have also added a new supplementary figure (**Fig S12**) depicting the temporal signal-to-noise ratio (tSNR) across voxels (p. 18):

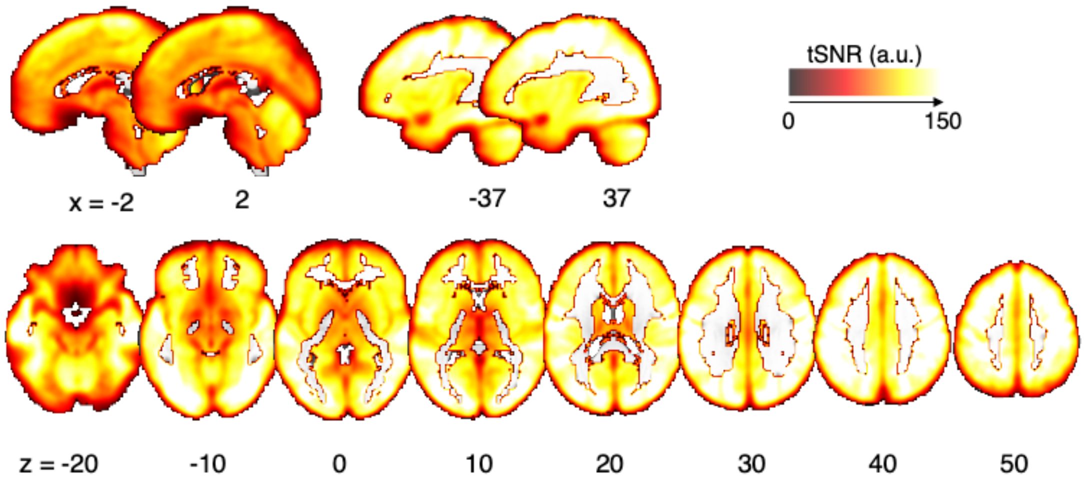

Fig. S12. Temporal Signal-to-Noise Ratio (tSNR) map. *We calculated the temporal signal-to-noise ratio (tSNR) using the TR-level data of the prediction task runs. We then averaged the tSNR values across runs and participants. The map shows the group average of the tSNR values.*

R3-13. *"The description of how the gradient map was created and what it means is not detailed enough - please consider expanding (also the legend of the associated figure), as currently it is difficult to follow and understand how this relates to their main findings."*

Response: We appreciate the reviewer's comment and agree that further clarification regarding the creation and interpretation of the gradient map will be beneficial. Below, we provide a detailed description of the analysis and its relevance to our main findings.

p. 12-13 (Results):
*This idea is further supported by the analysis using the principal gradient map [33] derived from the resting-state fMRI data of the participants in this study (**Fig. 4D**). The principal gradient axis, spanning from sensorimotor (unimodal) to association (transmodal) regions, has been proposed as a key functional organization in the human brain [33-36, 44]. This axis serves as an intrinsic coordinate system, supporting the progression of information processing from sensory inputs to higher cognitive functions. We used the BrainSpace Toolbox [45] to generate the principal gradient map based on resting-state data of the same participants (see Supplementary Text for more details). We divided the principal gradient into 10 or 100 bins (10-percentile and 1-percentile bins), creating binary masks for each gradient bin. We then calculated the overlap proportions between the supra-thresholded brain maps and these gradient bins [46, 47] (see Methods and Materials for more details). The results showed that the temporal dynamic patterns in the brain showed a clear transition from association to sensorimotor areas during the mediation of cue effects.*

p. 15 (**Fig. 4D** caption):
(D) (Left) The volumetric functional gradient map was derived from resting-state fMRI data of the current study participants (N = 56). This map shows the first connectivity gradient (i.e., principal gradient) represents a major functional axis spanning from association (transmodal) to sensorimotor (unimodal) brain systems (see Supplementary Text for more details). (Middle, Right) We divided the principal gradient into two sets of bins: one set comprising 10 bins using 10-percentile intervals (Middle) and another set of 100 bins using 1-percentile intervals (Right), creating binary gradient bin masks for each set. We then calculated the proportions of overlap between the thresholded brain maps and these gradient bin masks (see Materials and Methods for more details).

R3-14. *"Why did they choose three studies from one group to inform their ROI-based mediation analysis - might this lead to biased insights?"*

Response: Our ROI selections were guided by two key considerations. First, regions identified by Wager et al. [7] and Woo et al. [8] are well-established in the literature for their involvement in pain processing and modulation. For instance, the dorsal posterior insula (dpINS) is well-known for mediating the effects of stimulus intensity on pain [9] processing noxious stimuli [10], while the vmPFC and NAc are recognized for their role in self-regulation in healthy participants [8] and the transition from acute to chronic pain [11]. Second, we selected regions from Koban et al., [12], as their experimental manipulation closely aligns with that of the current study. Thus, even though the ROIs were extracted using three studies from one group, they include the regions well-studied across different research groups.

Reference

1. Senkowski D, Höfle M, Engel AK. Crossmodal shaping of pain: a multisensory approach to nociception. Trends in Cognitive Sciences. 2014;18(6):319-27. doi: 10.1016/j.tics.2014.03.005.
2. Seminowicz DA, Davis KD. Interactions of Pain Intensity and Cognitive Load: The Brain Stays on Task. Cerebral Cortex. 2006;17(6):1412-22. doi: 10.1093/cercor/bhl052.
3. Elkhetali AS, Vaden RJ, Pool SM, Visscher KM. Early visual cortex reflects initiation and maintenance of task set. NeuroImage. 2015;107:277-88. doi: https://doi.org/10.1016/j.neuroimage.2014.11.061.
4. Dale AM. Optimal experimental design for event-related fMRI. Human Brain Mapping. 1999;8(2-3):109-14. doi: https://doi.org/10.1002/(SICI)1097-0193(1999)8:2/3<109::AID-HBM7>3.0.CO;2-W.
5. Bartoshuk LM, Duffy VB, Green BG, Hoffman HJ, Ko CW, Lucchina LA, et al. Valid across-group comparisons with labeled scales: the gLMS versus magnitude matching. Physiology & Behavior. 2004;82(1):109-14. doi: https://doi.org/10.1016/j.physbeh.2004.02.033.
6. Rissman J, Gazzaley A, D'Esposito M. Measuring functional connectivity during distinct stages of a cognitive task. NeuroImage. 2004;23(2):752-63. doi: https://doi.org/10.1016/j.neuroimage.2004.06.035.

7. Wager TD, Atlas LY, Lindquist MA, Roy M, Woo C-W, Kross E. An fMRI-Based Neurologic Signature of Physical Pain. New England Journal of Medicine. 2013;368(15):1388-97. doi: 10.1056/NEJMoa1204471. PubMed PMID: 23574118.
8. Woo C-W, Roy M, Buhle JT, Wager TD. Distinct Brain Systems Mediate the Effects of Nociceptive Input and Self-Regulation on Pain. PLOS Biology. 2015;13(1):e1002036. doi: 10.1371/journal.pbio.1002036.
9. Atlas LY, Lindquist MA, Bolger N, Wager TD. Brain mediators of the effects of noxious heat on pain. PAIN®. 2014;155(8):1632-48. doi: https://doi.org/10.1016/j.pain.2014.05.015.
10. Segerdahl AR, Mezue M, Okell TW, Farrar JT, Tracey I. The dorsal posterior insula subserves a fundamental role in human pain. Nature Neuroscience. 2015;18(4):499-500. doi: 10.1038/nn.3969.
11. Baliki MN, Petre B, Torbey S, Herrmann KM, Huang L, Schnitzer TJ, et al. Corticostriatal functional connectivity predicts transition to chronic back pain. Nature Neuroscience. 2012;15(8):1117-9. doi: 10.1038/nn.3153.
12. Koban L, Jepma M, López-Solà M, Wager TD. Different brain networks mediate the effects of social and conditioned expectations on pain. Nature Communications. 2019;10(1):4096. doi: 10.1038/s41467-019-11934-y.
